# Cyanides, Isocyanides, and Hydrides of Zn, Cd and Hg from Metal Atom and HCN Reactions: Matrix Infrared Spectra and Electronic Structure Calculations

**DOI:** 10.1002/cphc.202100011

**Published:** 2021-08-13

**Authors:** Hongmin Li, Yetsedaw A. Tsegaw, Lester Andrews, Carl Trindle, Han‐Gook Cho, Tony Stüker, Helmut Beckers, Sebastian Riedel

**Affiliations:** ^1^ Institut fur Chemie und Biochemie Freie Universitat Berlin Fabeckstr. 34–36 14195 Berlin Germany; ^2^ Department of Chemistry University of Virginia 22904 Charlottesville Virginia USA; ^3^ Department of Chemistry Incheon National University 119 Academy-ro, Yeonsu-gu 22012 Incheon South Korea

**Keywords:** cyanides, electronic structure, Group 12, isocyanides, zinc

## Abstract

Zinc and cadmium atoms from laser ablation of the metals and mercury atoms ablated from a dental amalgam target react with HCN in excess argon during deposition at 5 K to form the MCN and MNC molecules and CN radicals. UV irradiation decreases the higher energy ZnNC isomer in favor of the lower energy ZnCN product. Cadmium and mercury atoms produce analogous MCN primary molecules. Laser ablation of metals also produces plume radiation which initiates H‐atom detachment from HCN. The freed H atom can add to CN radical to produce the HNC isomer. The argon matrix also traps the higher energy but more intensely absorbing isocyanide molecules. Further reactions with H atoms generate HMCN and HMNC hydrides, which can be observed by virtue of their C−N stretches and intense M−H stretches. Computational modeling of IR spectra and relative energies guides the identification of reaction products by providing generally reliable frequency differences within the Zn, Cd and Hg family of products, and estimating isotopic shifts using to ^13^C and ^15^N isotopic substitution for comparison with experimental data.

## Graphical abstract

**Matrix infrared spectra**: Outline of the reactions that occur with laser ablated Hg atoms and HCN molecules during condensation in excess argon at 5 K and subsequent annealing to allow diffusion and reaction of H atoms with the HgCN and HgNC primary reaction products to form their hydrides.

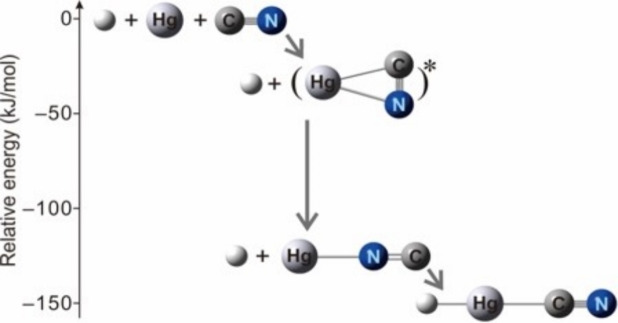

## 1 Introduction

For investigation of novel cyanide and isocyanide molecules with infrared (IR) spectroscopy, we have produced by laser ablation Group 2, 3, 4, 7, 8 and 13 metal atoms as well as the Ce, Nd, Th, and U atoms. We have observed their reactions with cyanogen and U with hydrogen cyanide, and Zn with oxygen and hydrogen mixtures.[^1^, ^2^, ^3^, ^4^, ^5^, ^6^, ^7^, ^8^, ^9^, ^10^, ^11^, ^12^, ^13^, ^14^, ^15^, ^16^, ^17^, ^18^, ^19^, ^20^]

Cyanogen generally reacts with metal atoms to form dicyanides or even tetracyanides.[^10^, ^11^, ^12^, ^13^, ^14^, ^15^, ^16^, ^17^, ^18^, ^19^] Our work with the Group 2 metal atoms and HCN or with (CN)_2_ provided IR spectra for the MNC isocyanides with no obvious amount of the cyanide isomer MCN.^[18]^ The laser ablated Zn atom reaction with (CN)_2_ in excess argon during condensation at 5 K produced ZnNC with an intense N−C stretching frequency of 2074.6 cm^−1^: the observed effects of ^13^C and ^15^N substitution were consistent with the isocyanide structure.^[18]^ The ZnNC isomer could also be produced by UV irradiation of Zn atoms and (CN)_2_ co‐deposited in solid argon.^[18]^ The N−C stretch for the analogous CdNC molecule was observed at 2069.7 cm^−1^ in experiments reacting Cd and (CN)_2_,^[20]^ and the analogous higher energy HgNC molecule is produced here from HCN and Hg ablated from a dental amalgam target.^[9]^ This novel use of a dental amalgam ablation target adds more to the known chemistry for mercury.

The Largo group has performed a theoretical investigation of the structure, bonding, and isomerization barriers for the first‐row transition metal cyanides and isocyanides.[^21^, ^22^] Of particular interest to us, they have predicted a modest dissociation energy for ZnNC to Zn+CN (ca. 50 kcal/mol) and a low isomerization barrier for ZnNC to ZnCN (ca 5 kcal/mol) which are supported by the trends reported here.

Infrared detection of the weakly absorbing cyano species is more challenging, which is why we used here the more reactive HCN precursor. A previous Microwave spectroscopic investigation revealed that ground state ZnCN was formed from a mixture of zinc vapor and cyanogen gas in a DC discharge, and provided its bond length values.^[23]^ Later work using Zn(CH_3_)_2_ as a source of zinc and hydrogen atoms with cyanogen in the discharge produced a related molecule, HZnCN, which was also identified from its microwave spectrum,^[24]^ and the hydride absorptions here increase on annealing to allow diffusion and reactions of H atoms first trapped in this matrix isolation investigation. The use of HCN as the reagent provides H atoms to react with cyanides and isocyanides to form their monohydrides.

Reported calculations provide helpful information on experimentally known and hypothesized (H, M, C, N) systems. For example, vibrational frequencies, energies, and structures are obtained directly. Andrews and Cho^[18]^ explored the reaction of Zn with NCCN and employed a density functional (B3LYP) method and a coupled‐cluster expansion CCSD(T) method with a large Dunning basis (aug‐cc‐pVTZ) to describe isomers of general form (C,N)Zn_k_ (C,N). They reported the impact of ^13^C isotopic substitutions on vibrational frequencies, and for the CCSD and DFT calculations, supplied the vibrational intensities. The latter calculation showed that the C−N stretch is much more intensely absorbing for Zn−N−C than for Zn−C−N, while ZnCN is more stable than ZnNC by 20.7 kJ/mol at CCSD(T) level.

Here follows the complementary investigation of Zn, Cd and Hg atom reactions with HCN, which are more productive than the reactions with NCCN studied earlier.[^18^, ^20^] Additional information was obtained with the H^13^CN reagent and the resulting isotopic shifts and isotopic frequency ratios became useful diagnostics. Our goal using the matrix isolation technique was to produce the zinc, cadmium, and mercury cyanides MCN and isocyanides MNC in these metal atom reactions with HCN, and to measure their argon matrix infrared spectra. As anticipated from previous work we observed a 5 fold‐stronger signal for ZnNC produced from the HCN reagent than for the Zn reaction with cyanogen.^[18]^ We have found HCN to be more reactive than NCCN, which gives us a much better possibility for observing the ground state ZnCN isomer with the lower IR intensity for this essentially covalent ZnC−N bond vibration. In addition, the easy production of H atoms from HCN allows formation of the related HMCN and HMNC monohydride molecules. In the final analysis the use of two different CN bearing precursors to make the same new molecules provides a better understanding of their spectra and quite different bonding properties. This gives us the unique opportunity to investigate these new (Ar)_n_‐CN^+^ complexes where n could be 1,2,3,4, for example.

## Experimental Methods

Laser‐ablated Zn and Cd and Hg atoms were reacted with the HCN and H^13^CN molecules in argon host gas during their deposition at 5 K using a closed‐cycle helium refrigerator (Sumitomo Heavy Industries, RDK‐205D) inside of a self‐made vacuum chamber, which has been described in detail previously.^[9]^ Hydrogen cyanides were synthesized by reacting KCN with dilute H_2_SO_4_ and drying the HCN gas by trap to trap distillation. The H^13^CN was made from K^13^CN (99 %) in the same way. The 1064 nm fundamental of a Nd:YAG laser (10 Hz repetition rate with 10 ns pulse width), and a pulse energy up to 55 mJ) was focused onto different rotating metal targets. An amalgam previously in common use for dentistry served as a target for Hg. This amalgam, which was composed of about 50 % Hg and 50 % of the more noble Cu, Ag and Sn metals by weight, was pressed into a disc of about 13 mm diameter and 4 mm thick, and glued onto a sample holder for rotation (picture in supporting information, SI). This method produced ample Hg atoms for our work.[^9^, ^17^] Infrared (IR) spectra were recorded at 0.5 cm^−1^ resolution on a FTIR vacuum spectrometer (Bruker Vertex 70v) equipped with a transfer optic and Mid‐IR MCT detector (4000–450 cm^−1^). Matrix samples were annealed at different temperatures and cooled back to 5 K, and selected samples were irradiated in the ultraviolet (UV) by a medium pressure mercury arc street lamp (175 W):[^1^, ^2^, ^3^, ^4^, ^5^, ^6^, ^7^, ^8^, ^9^, ^10^, ^11^, ^12^, ^13^, ^14^, ^15^, ^16^, ^17^, ^18^, ^19^, ^20^] Observed and calculated vibrational frequencies are reported here in cm^−1^ units.

## Computational Methods

We conducted density functional, and coupled‐cluster calculations primarily with Gaussian 16 software.^[26]^ Frequencies, absorption intensities, bond lengths, and energies were obtained with the B3LYP hybrid density functional^[27]^ using the aug‐ccpVTZ basis set^[28]^ for C, N, and Zn and the aug‐cc‐pVTZ‐pp^[29]^ basis for Cd and Hg. The structures obtained by geometry optimization were verified to be relative minima in energy by vibrational analysis. The calculated harmonic frequencies are reported here unscaled. Such DFT calculations predict vibrational frequencies 2 to 10 cm^−1^ higher than observed in the argon matrix for metal compounds.^[10]^ These comparisons between model and data suffer from (1), the accuracy of the theoretical method involved, (2) the usually higher value of harmonic (calculated) relative to anharmonic (observed) frequencies, and (3) the common matrix shift to lower frequencies owing to the generally weak dispersive interaction of the matrix host with the guest molecule.

Separate calculations were done with CCSD, to provide absorption intensities not available in the Gaussian CCSD(T) implementation. Zero‐point energies derived from vibrational calculations are included in the energy reports. Additional calculations were done with the Molpro system^[30]^ to address an issue that came up in the modeling of the metal cyano systems.

## 2 Zinc Studies

### 2.1 Spectroscopic Results and Discussion (Zinc Systems)

**Figure 1:**
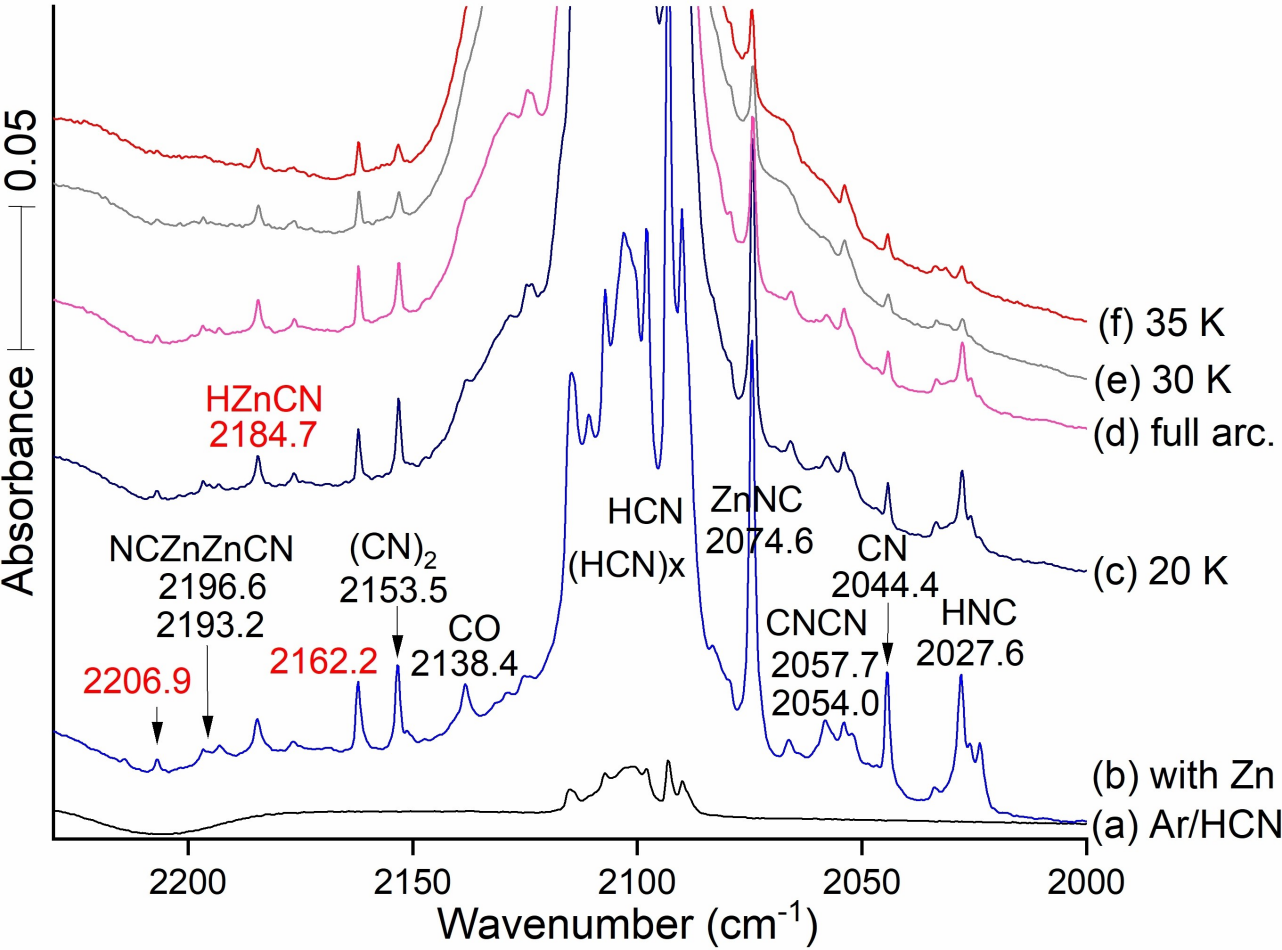
A series of spectra obtained from Zn and HCN in argon. (a) The black trace was taken after a 20 min deposition of the Ar containing 1 % HCN sample. The next spectrum (b) in light blue was recorded of the reaction products from after laser ablated Zn and 1 % HCN were deposited together in argon at 5 K for 120 min. The dark blue spectrum (c) was recorded after annealing to 20 K. The magenta spectrum (d) followed full Hg arc photolysis for 20 min. The gray spectrum (e) and the red trace (f)  were taken after annealing cycles to 30 and 35 K followed by cooling back to 5 K. Black numbers refer to transitions previously identified; red numbers refer to new observed absorptions. The weak 2206.9 band is not identified here. Now we focus on the new reaction product molecules which are the subject of this research.

Figure 1 displays infrared spectra for zinc and hydrogen cyanide reaction products. Many vibrational bands are labeled; red numbers identify new data. Black numbers in the spectra identify absorption bands observed in our previous work using laser ablated zinc and the (CN)_2_ reagent.^[18]^ Spectra begin with (a) the deposition of 10 mbar of argon gas from the 1.1 L vacuum manifold containing 1 % HCN[^1^, ^2^] in argon and continue through stages of processing scans (b‐f).

### 2.2 Non‐Metal Systems

Matrix spectra of HCN and its oligomers were first measured with prism, filter, and grating spectrometers.^[31]^ King and Nixon report signals for HCN in argon at 3303.3 (H−CN stretch), 2093 (C−N stretch) and 720.2 cm^−1^ (bend).^[31]^ The band at 2027.6 cm^−1^ was assigned to the HNC isomer by Milligan and Jacox, who produced the signal by UV photolysis of CH_3_N_3_ in solid argon and then by the vacuum ultraviolet photolysis of HCN during deposition in excess Argon.^[32]^ A signal at 2046 (in an argon matrix using an older instrument) was attributed to the stretch for CN radical by Milligan and Jacox;^[33]^ our recent value using FTIR is 2044.4 cm^−1^.

Modern FTIR instruments provide inherently more accurate values compared with the earlier numbers,[^31^, ^32^, ^33^] especially toward higher frequencies for example, we measured the C−H stretch of HCN at 3305.5 cm^−1^, (cf. 3303.3 for King and Nixon^[31]^). The H−C−N bending mode at 720.9 cm^−1^, (cf. 720.9^[31]^) and its overtone at 1426.4 cm^−1^ were found in our 0.2 % (1/500) HCN sample in argon. Abbate and Moore observed the bands at 720.9, and 1425.4 cm^−1^ previously using a more dilute 1/5030 argon sample^.[34]^ We consider that our values are accurate within about ±0.2 cm^−1^ but there are a number other factors which contribute to uncertainty such as guest concentration in the matrix, band width, substrate window temperature, and the resulting rate of sample deposition. Another consideration is local matrix site splittings: liner molecules such as CNCN and OCO have antisymmetric fundamentals that are split by the matrix. These will be shown later in Supporting Information (SI), and the major matrix site splitting bands for CNCN are given in Table 1.

**Table 1:** Frequencies [cm^−1^] observed from reactions of Zn atoms with H^12^CN and H^13^CN during co‐deposition in excess argon at 5 K. Bold type marks the major trapping site. Common absorptions for non‐metal species (CN)_2_, CNCN, HNC and CN are listed first. Frequencies for zinc‐bearing systems follow, which have counterparts in all the Group 12 metal experiments. “da”=decreased intensity upon annealing.

Non‐Metal Species A				
**H^12^CN**		**H^13^CN**	12/13 freq. ratio, shift	assignment
2294.9		2241.7	1.02373, 52.3	sym CNCN mode
2153.5		2097.8	1.02655, 55.7	(CN)_2_
2138.4		2091.5	1.02252, 46.9	CO
2097.9, **2093.1**,2089.9		2063.9, **2060.1**, 2056.8	1.01602, 33.0	HCN matrix sites
2100		2069	1.01498, 31	(HCN)_x_
2054.2,2053.7		2017.7,2109.4	1.01809, 34.5	asym CNCN&site
2044.4		2001.9	1.02123, 42.5	CN
2027.6, 2023.5 (da)		1984.6, 1980.6	1.02167, 43, 42.9	HNC and site variants

Zinc‐containing molecules B: cyano and isocyano species				
^12^C^14^N	^13^C^14^N	Isotopic ν Ratio	Computed ν	Assignment
2184.7	2136.2	1.02270	2198	HZnCN
2162.2	2114.9	1.02236	2177	ZnCN
2097.8	2060.1	1.01830	2102	HZnNC
2074.6	2034.0	1.01996	2083	ZnNC^[18]^
2196.0	2151.3	1.02078		N‐CZnZnC−N^[18]^
2097.7	2058.3	1.01914		C−NZnZnN‐C^[18]^
2200.5	2153.9	1.02164		N−CZnC−N^[18]^
2097.0	2057.5	1.01920		C−NZnN−C^[18]^
1996.0	1996.0	1.00000		H−ZnCN
1972.1 look at 2023.9	1972.1 2023.9	1.00000		H−ZnNC
^**12**^ **C^14^N**	^**12**^ **C^15^N**			
2162.2	2131.7	1.01431	2177	ZnCN
2074.6	2041.0	1.01646	2083	ZnNC^[18]^

Zinc‐containing molecules B. continued hydrides				
^1^H	^2^D	Isotopic ν Ratio	Computed ν	Assignment
{1870.2}	{1870.2}			ZnH_2_^6^
{1493.9}	{1493.9}			ZnH ^6^
1889.4	1371.6	1.37752		ZnH_2_ ^[35]^

Figure 1 shows our spectrum of HCN in argon first then more of the same sample co‐deposited with Nd‐YAG fundamental laser ablated Zn. For spectrum a, the stronger sharp band on the right side at 2093.1 cm^−1^ is due to isolated HCN,[^31^, ^33^] and the strong broad band at 2103 cm^−1^ arises from HCN dimers and higher polymers labeled (HCN)_x_. The remaining spectra in Figure 1(b) through (f) illustrate absorptions for a sequence of zinc reaction products in the 1 % HCN sample in argon. The spectrum (b) followed the co‐deposition of 120 mbar more of argon with HCN from a constant 1.1 Liter volume, and laser ablated zinc using 50 % of maximum laser energy for 2 h. Cyanogen provides the band at 2153.5 cm^−1^ presumably from the dimerization of CN radicals. The sharp band at 2054.0 cm^−1^ shown in spectrum (b) of Figure 1 corresponds to a feature found in our studies of transition metals, uranium, and thorium with cyanogen.[^10^, ^11^, ^12^, ^13^, ^14^] This is due to the CNCN photoisomer of cyanogen.^[18]^ In the current work with HCN, it appeared at 2053.7 cm^−1^. Such small differences are a part of the matrix isolation experiments. The sharp band at 2954.0 on (b) in Figure 1 is favored by metals with cyanogen, and it is assigned to the CNCN photoisomer of NCCN.^[18]^ Both matrix sites at 2054.0 and 2057.7 are observed with the larger product yield in Figure 1. More examples of matrix site splittings are found in our ref. [18].

#### 2.2.1 Appearance of ZnCN, ZnNC, HZnCN and HZnNC

**Figure 2:**
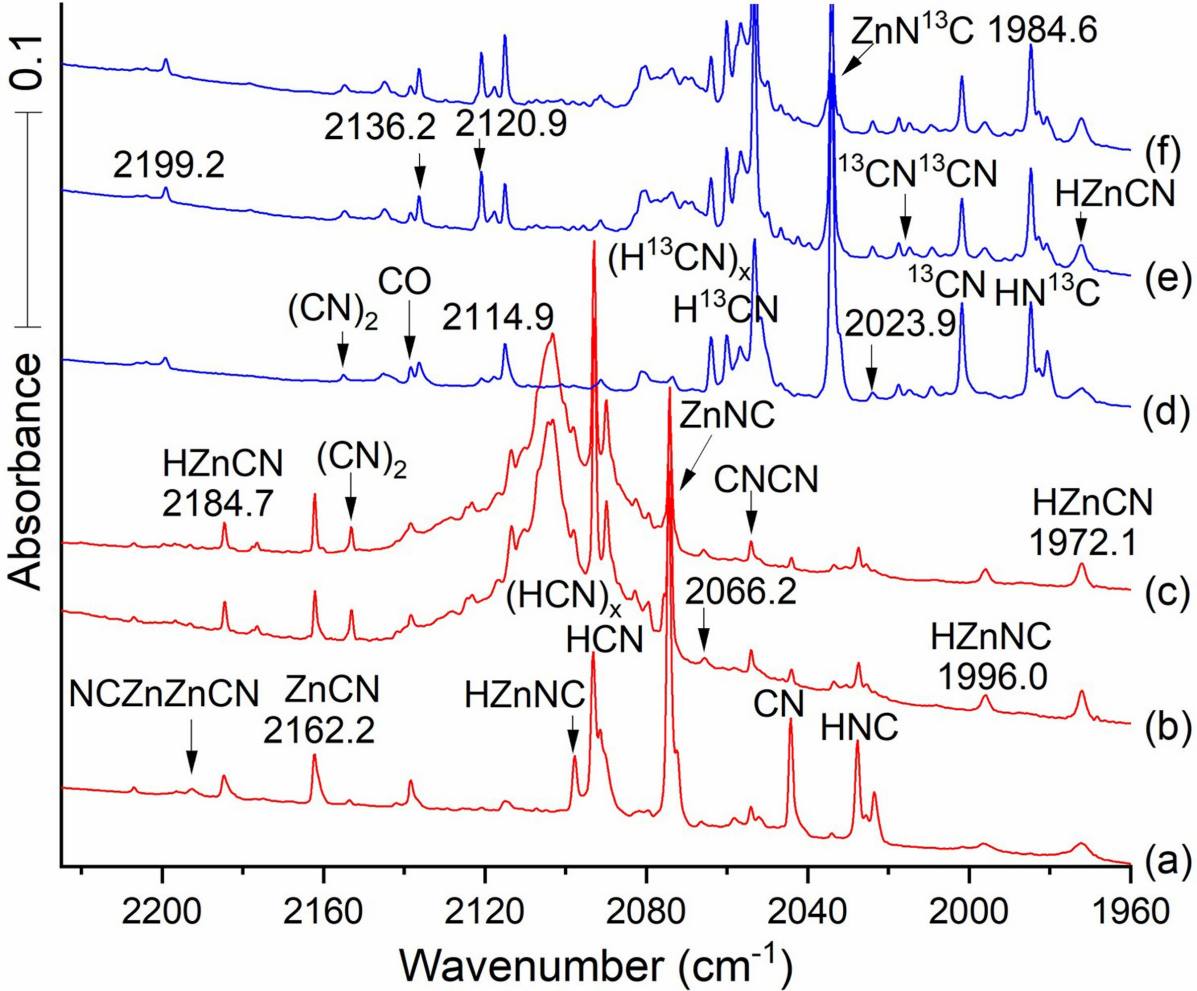
Infrared spectra of the reaction products from laser ablated Zn co‐deposited with 0.2 % HCN in Ar. Red spectra (a–c) from co‐deposition of Zn atoms and with H^12^CN; blue spectra (d–f) from co‐deposition of Zn atoms and H^13^CN in argon at 5 K. Spectra (a) and (d) were measured after deposition for 120 min, (b) and (e) followed annealing to 20 K. (c) and (f) after full arc UV irradiation for 20 min. *Notice that the HZnNC and HZnCN bands increase on annealing to 20 K, scans (b) and (e) relative to scans (a) and (d)*.

**Figure 3:**
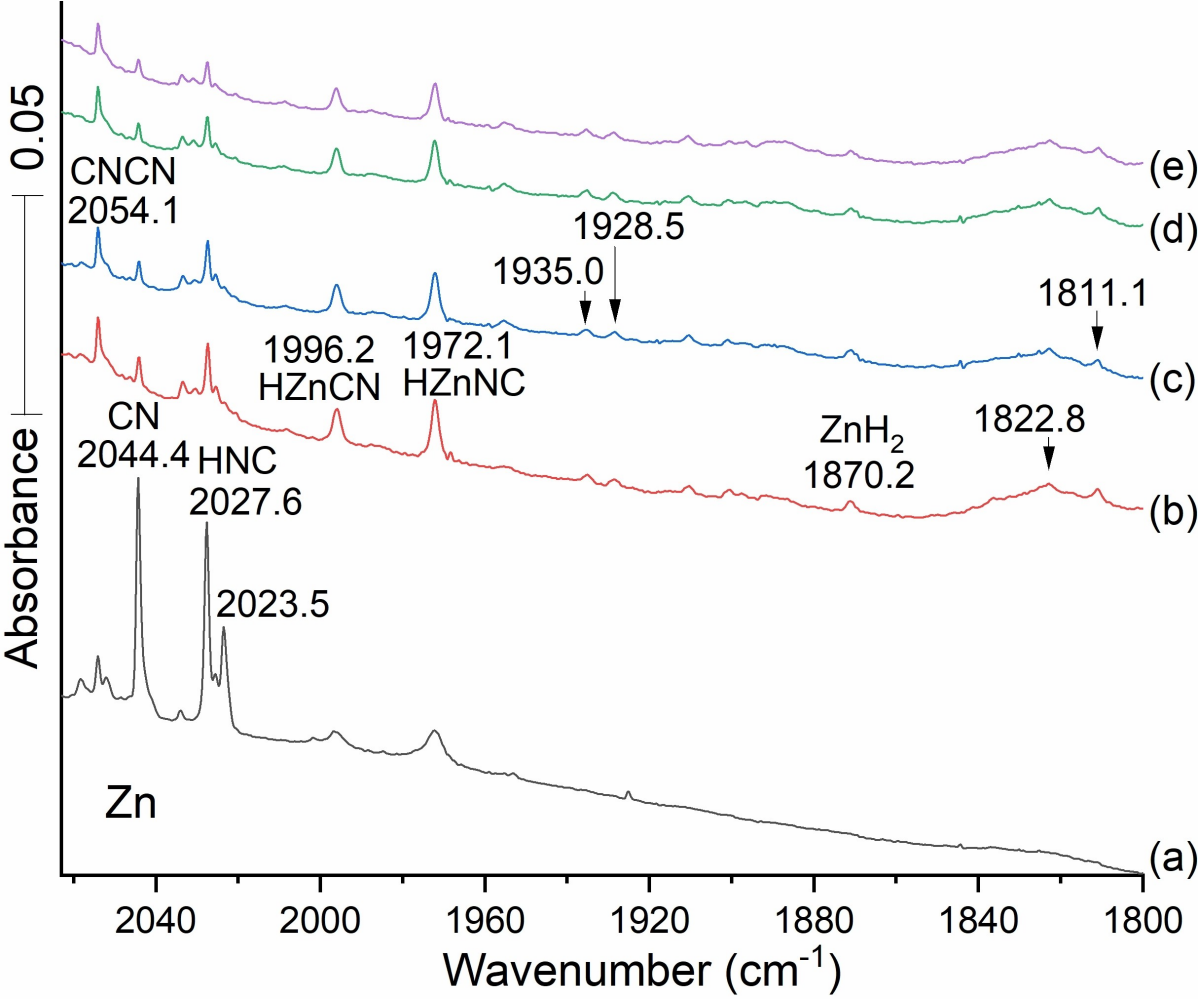
The infrared spectra between 2060 and 1800 cm^−1^ for the reaction products from laser ablated Zn co‐deposited with 0.2 % HCN in argon at 5 K. Spectra (a–e) are observed after the following steps: (a) deposition for 120 min, (b) annealing to 20 K and cooling back to 5 K, (c) full Hg arc irradiation for 20 min, (d) annealing to 30 K, and (e) annealing to 35 K.

The CN radical[^1^, ^2^, ^18^] presents a strong absorption at 2044.4 cm^−1^ on Ar/Zn/HCN sample deposition (Figure 1, spectrum b). Upon annealing to 20 K (spectrum c) the CN signal decreases by about 90 % while the 2153.5 cm^−1^ (CN)_2_ band increases only slightly because other, namely metal atom, reactions with CN, are more favorable than its dimerization. Weak bands are found in Figure 1 spectrum (b) at 2196.6 and 2193.2 cm^−1^, in excellent agreement with the site splittings first reported for the dizinc molecule NCZnZnCN.^[18]^ Important newly discovered (red‐type) bands lie at 2162.2 and 2184.7 cm^−1^ in Figures 1 and 2 where they are labeled with the above numbers and the latter is also labeled HZnCN since there is room in the spectrum for this. The 2184.7 band increases slightly on annealing to 20 K (shown in spectrum c) while the 2162.2 band decreases slightly: These bands are only 22.5 cm^−1^ apart, which suggests a common CN group, with stretching frequency affected slightly differently by the Zn‐ or HZn‐ substituents. The 2162.2 band assigned here to ZnCN, which readily adds H upon annealing to form HZnCN. The very strong sharp 2074.6 band due to ZnNC, which also adds H, to form HZnNC, upon annealing (seen in Figures 2 and 3). The latter bands are 110.1 cm^−1^ apart, which is about the difference we find here between related cyano and isocyano stretching modes. The carbon‐13 counterparts for these bands are 2136.2 and 2060.1 which define 12/13 frequency ratios of 1.02270 and 1.01830 that reveal differences between cyano and isocyano stretching modes (the higher value is characteristic of a C−N stretching mode and the lower value comes from an M−N−C stretching vibration. We observed two other frequencies for these two molecules, lower at 1996.2 and 1972.1, which have the same values for carbon 13: This and our calculations show that these are the Zn−H stretching modes, respectively, for the two hydride product molecules.

The ablation process produced substantial vacuum UV light, leading to photolysis of HCN^[10]^ during sample deposition. This process also provides the H atoms required for radical‐radical addition of H to the ZnCN cyanide and ZnNC isocyanide isomers above to make the new HZnCN and HZnNC hydrides, which are assigned here. This radical‐radical combination is highly exothermic (Energetics are in Table S1).

#### 2.2.2 Isotope Effects

Figure 2 compares vibrational features for the normal and ^13^C labeled species including HCN, HNC, and ZnNC. Table 1 provides more details for ZnCN, ZnNC, HZnCN and HZnNC. The isotope effect on the C−N stretch is larger for the cyano systems than the isocyano systems, suggesting that displacement of the labeled Carbon is greater in the normal mode for ZnCN than for ZnNC.

The weak bands at 2054.0 and 2057.7 in Figure 1 are matrix site splittings for CNCN and they are more intense in experiments with the cyanogen precursor, which is isoelectronic with CNCN.^[18]^ Space limitations may determine what can be used for labels in a particular figure. Two matrix site bands are observed for NCZnZnCN in Figure 1 at 2193.2 and 21966. More of these bands were observed at higher HCN concentration in Figure 1 than at lower HCN concentration in Figure 2.

Our previous work using a statistical mixture of cyanogen with ^14^NCC^15^N provided the sharp above 2074.6 cm^−1^ band for ZnNC and a similar band at 2041.0 which exhibited the same increase on annealing and decrease on photolysis as the first band. The ^14^N/^15^N isotopic frequency ratio 1.01646 is less than the ^12^C/^13^C isotopic frequency ratio 1.01986 owing to its lower reduced mass.^[18]^ Thus the strong 2074.6 band is due to the isocyanide ZnNC and the 33.6 cm^−1^ lower band at 2041.0 is the right amount for the Zn^15^NC shift and 14/15 ratio 1.01646.^[18]^ Another band in the statistical sample spectrum at 2131.7 was 90.7 cm^−1^ higher than the 2041.0 band for Zn^15^NC, which is appropriate for its cyanide isomer ZnC^15^N as assigned.^[18]^

Figure 2 also contains the new band at 2184.7 cm^−1^ in the neighborhood near the ZnC−N stretch, 22.5 cm^−1^ higher than the new 2162.2 band assigned above to ZnCN. The 2184.7 cm^−1^ band has its ^13^C counterpart at 2136.2 cm^−1^, just below the CO band, which gives a healthy 48.5 cm^−1^ shift and a 1.02270 ^12^C/^13^C frequency ratio. This shift and ratio closely match the 47.3 cm^−1 13^C shift and ratio 1.02236 associated with the 2162.2 cm^−1^ band now assigned to ZnCN. This indicates that the 2184.7 cm^−1^ and 2136.2 cm^−1^ bands are associated with a ZnC−N functional group. HZnCN is the clear candidate to explain the 22.5 shift for the 2184.7 band. The sharp 2097.8 band is due to HZnNC owing to the large yield of the ZnNC radical for favorable reaction with H atoms.

Figure 3 shows that the two lower frequency monohydride absorptions clearly increase on annealing which promotes H atom reactions, but the more strongly absorbing HNC band at 2074.6 cm^−1^ declines in intensity on photolysis while the weaker absorption at 2162.2 cm^−1^ increases upon photolysis. The key here is the lower carbon ^12^C/^13^C frequency ratio, i. e. (2074.6/2034.22)=1.01986, is diagnostic of the isocyano stretch, to be compared with the higher ratio for the 2162.2 band, (2162.2/2114.9)=1.02236 characteristic of the cyano stretch. The 2184.7 band is just above HCN, which is observed in Figure 2 using lower reagent concentration, and this band is due to HZnCN. Figure 3 also features two additional new IR bands in the Zn−H stretching region at 1996.2 and 1972.1 both above ZnH_2_ at 1870.2 cm^−1^ (1889.43, gas phase^[35]^). These are associated with an annealing step, which allows trapped H atoms to diffuse and react with open shell molecules in the matrix. The assignment of these modes as H−Zn stretches is assured by their zero carbon‐13 shifts. The same frequency bands are observed in scans a,b,c, also with HCN, as in d,e,f. The experimental frequency separation between the H−Zn and C−N mode at 2184.7 and the ZnC−N mode at 2162.2 cm^−1^ (22.5 cm^−1^) is similar to the frequency separation between the calculated HZnN−C mode at 2097.8 cm^−1^ and the ZnN−C mode at 2074.6 (that is, 23.2 cm^−1^). These two isocyano absorptions decrease on UV photolysis as the corresponding lower energy cyano signals increase. This parallel allows us to confirm the assignment of both the absorptions at 2184.7 and 1996.2 cm^−1^ to HZnCN's cyano C−N isomer and H−Zn motions and the two bands at 2097.8 and 1972.1 cm^−1^ to HZnNC's isocyano Zn−N−C and H−Zn stretches.

Table 1 collects the numerical values of the observed C−N frequencies, the isotopic frequency ratios of ^12^C and ^13^C species, and assignments for the observed frequencies. Cyanogen and its isomer CNCN, CO, CN, HCN, and HNC have been identified. Frequencies have been assigned to Zn cyanide and isocyanide and to the hydrogen addition hydrides as well. Data for NCZnZnCN and ZnH_2_ from previous work are also included.^[18]^ The HNC and HCN isomers are related by photolysis.^[26]^ The cyano stretch for HZnCN is 22.5 cm^−1^ higher than the cyano stretch in ZnCN; The isocyano stretch for H−ZnNC lies 23.2 cm^−1^ higher than the isocyano stretch in ZnNC. This suggests that the formation of the hydride affects the (C−N) groups in small and similar ways. The observed blue shifts for the hydrides are slightly higher than the CCSD computed values for the cyano, 6.2 and isocyano 14.4 cm^−1^. The ^12^C/^13^C isotopic frequency ratios for Zn‐bearing species (also shown in Table 1) fall into two sets, (A) greater than 1.021 for cyano systems and (B) smaller than 1.021 for isocyano species. This generalization applies to systems with one or two Zn atoms and two (iso)cyano fragments, according to previous work.[^6^, ^18^] We observed that the band at 1972.1 cm^−1^, assigned to the H−ZnCN mode for HZnCN, lies 101.9 cm^−1^ higher than the Zn‐asymmetric stretch for ZnH_2_ at 1870.2 (in solid argon). More 12/13 frequency ratios are in Table 1 below.

The ^15^N isotopic data are from statistical NC=C^15^N. We confine discussion to the strongest bands of ZnNC and ZnCN.

Figure S2 spectra show the effect of increasing laser energy by 20 %. This boost increased the intensity of most of the product absorptions, particularly the CN and HNC bands produced by ablation photochemistry.

### 2.3 Estimate of Abundances for Zinc Cyanide and Isocyanide

We measured absorbances A for the 2074.6 cm^−1^ ZnNC and 2162.2 cm^−1^ ZnCN bands in 0.2 % and 1.0 % concentration experiments (Figures 2 and 1). In the deposit with 1 % reagent we find the band absorbance measured is 0.1344 for the 2074.6 cm^−1^ band while the value for the 2162.2 cm^−1^ band is 0.02364. We can obtain an estimate of the relative abundance of cyano and isocyano species from the computed absorption intensities (see next section). Normalize by dividing the absorbances by the CCSD computed intensities for these two modes: 276 and 26 km/mol, respectively, we find a measure of abundance estimated for the 2074.6 isocyano band to be 4.9×10^−4^ and for the 2162.2 cm^−1^ cyano band to be 9.1×10^−4^. For the 0.2 % reagent, the corresponding values are 5.9×10^−4^ and 8.7×10^−4^. These numbers show that our matrix traps 2× more of the more stable ZnCN cyanide isomer than the 17.3 kJ/mol (CCSD) higher energy ZnNC isocyanide. The same treatment can be applied to the hydrides HZnNC and HZnCN, whose 2097.8 and 2184.7 cm^−1^ bands are 5/3 in relative absorbance in the 0.2 % experiment. (We could not measure the 2097.8 cm^−1^ band in the 1 % HCN experiment owing to interference from HCN polymers). The relative populations are roughly 6 : 1 in favor of the more stable HZnCN. Only the extremely high intrinsic infrared intensities for the isocyano systems makes their presence prominent in the infrared spectra.

### 2.4 Computational Modeling (Zinc Systems)

One of the most demanding, and for predominantly single‐determinant states one of the most reliable electronic structure methods is the coupled cluster expansion. Usually the CCSD(T) form, expressed in an extended basis set such as aug‐cc‐pVTZ approaches chemical accuracy in energy terms and estimates harmonic vibrational frequencies within at most a few tens of wave numbers. Computational modeling provides energetics and structures. We defer discussion of these properties to a later comparison of all three metal systems. Here we focus on the computed vibrational frequencies including isotope effects for the Zinc bearing molecules. We report both CCSD(T) and CCSD results since absorption intensities are available in the Gaussian 16 implementation for CCSD, and CCSD provides a comparison point for the CCSD(T) vibrational frequencies.

Vibrational frequencies for HCN and H^13^CN reaction products with of Zinc using the CCSD and CCSD(T) methods.

Table 2 contains computed vibrational frequencies for isotopic variants of Zinc systems. The computed ^12^C/^13^C frequency ratios clearly distinguish cyano from isocyano systems, and are consistent with the experimental ratios. Specifically, the observed Zn^12^CN/Zn^13^CN frequency ratio (2162.2/2114.9) is 1.0224; the ratios calculated from the CCSD and CCSD(T) frequencies are 1.02271 and 1.02313, in close agreement. The consistency confirms assignment of the new observed 2162 cm^−1^ band to the ZnC−N mode. The theoretical (harmonic) frequency ratios are slightly larger than the experimental values, which are due to their anharmonicity.

**Table 2:** Coupled cluster calculations for sigma stretching modes in Zinc systems using G‐16.

2A: CCSD	ν1 σ (C, N) (Intensity)	Isotopic Ratios^[a]^	ν2 σ (H)Zn‐(C, N) (Intensity)	ν4 σ H−Zn (Intensity)	ν1–ν4
ZnCN	2260.3 (26)		418.8 (58)		
Zn^13^CN	2210.1 (23)	1.02236	413.7 (57)		
ZnC^15^N	2227.6 (28)	1.01457	412.8 (56)		
ZnNC	2148.2 (276)		472.1 (81)		
ZnN^13^C	2106.0 (276)	1.02003	464.2 (78)		
Zn^15^NC	2112.7 (258)	1.01680	465.4 (79)		
HZnCN	2266.5 (25)		467.8 (39)	2008.0 (85)	258.5
DZnCN	2266.5 (25)		466.7 (39)	1431.8 (48)	
HZn^13^CN	2215.7 (23)	1.02293	462.3 (38)	2008.0 (85)	
HZnC^15^N	2234.4 (27)	1.01707	461.0 (38)	2008.0 (84)	
HZnNC	2162.6 (264)		521.2 (56)	2027.4 (62)	135.2
DZnNC	2162.6 (261)		519.7 (55)	1446.1 (38)	
HZnN^13^C	2120.9 (265)	1.01966	513.3 (54)	2027.4 (59)	
HZn^15^NC	2126.3 (249)	1.01707	515.0 (55)	2027.4 (61)

2B: CCSD(T)	ν1 σ	Isotopic Ratios^[b]^	ν2 σ (H)Zn‐(C,N)	ν4 σ H−Zn	ν1–ν4
ZnCN	2277.6		416.4		
Zn^13^CN	2226.1	1.02313	411.4		
ZnC^15^N	2243.7	1.01511	410.5		
ZnNC	2083.6		466.6		
ZnN^13^C	2042.9	1.01992	459.7		
Zn^15^NC	2049.0	1.01689	461.0		
HZnCN	2196.0		467.5	2010.8	185.2
DZnCN	2196.0		466.3		
HZn^13^CN	2146.6	1.02301	462.0		
HZnC^15^N	2165.1	1.01427	460.7		
HZnNC	2099.3		519.1	2028.2	71.1
DZnNC	2099.3		517.7		
HZnN^13^C	2059.1	1.01952	511.2		
HZn^15^NC	2063.9	1.01715	513.0

[a] (^12^C/^13^C) or (^14^N/^15^N). [b] (^12^C/^13^C or ^14^N/^15^N).

The naturally occurring abundance of ^15^N in ^14^NC=C^15^N gave ZnC^15^N and Zn^15^NC absorptions at 2131.7 and 2041.0 cm^−1[18]^ as shown in Table 1. The ratio of CN stretching frequencies for ZnC^14^N/ZnC^15^N isotopic substitution is 1.01457 according to CCSD calculations, 1.01511 by CCSD(T). The observed ^14^N/^15^N frequency ratio for ZnNC is 1.01996 while the computed values are 1.02003 and 1.01992 for CCSD and CCSD(T) respectively. The observed ^14^N/^15^N frequency ratio for ZnNC is 1.01680; CCSD and CCSD(T) values are 1.01680 and 1.01689, respectively. Comparison with the ZnCN ratios suggests that there is more N motion and less C movement in the isocyanide than in the cyanide. A more thorough discussion of this statement is covered in Supplementary Information. The isotopic frequency ratios enable definitive assignments for these two isomers.

The parallel between (C−N) stretching isotopic frequency ratios in the Zn(C, N) and HZn(C,N) species is clear. For HZnCN, the observed ^12^C/^13^C ratio, 1.02270, matches CCSD and CCSD(T) values, 1.02293 and 1.02301, respectively. The corresponding observed ^12^C/^13^C value for HZnNC, 1.01830, is to be compared with 1.01966 and 1.01952 computed values. The CCSD(T) computed ^14^N/^15^N ratio for ZnNC is 1.01689, while the corresponding ratio for ZnCN is 1.01511; the values for HZnNC and HZnCN are 1.01715 and 1.01427, respectively. The greater values for the isocyano systems suggest that the N atom displacement is greater in the isocyano systems. A parallel argument indicates that the C atom displacement is greater in the cyano systems than in the isocyano systems.

It is interesting to examine the computed frequencies associated with HZnCN and HZnNC with those of ZnCN and ZnNC. The isocyano and Zn−H stretching modes for HZnNC are computed with CCSD as 2162.6 cm^−1^ (intensity 264 km/mol) and 2027.4 (intensity 62) while the cyano and Zn−H stretches are 2266.5 and 2008.0 cm^−1^ (Table 2A, below). The N−C stretch in HZnNC is computed by CCSD to be more strongly absorbing than the CN stretch by 264/62=4.3 times. Frequencies obtained using CCSD(T) are 2099.3 and 2028.2 cm^−1^ for the HZnN−C and H−Zn stretching modes, respectively. CCSD(T) calculations on HZnCN yield values of 2196.0 for C−N and 2010.8 for H−Zn stretches (no intensities are produced). The difference between computed cyano and isocyano stretches (96.7 cm^−1^) is consistent with the widely observed 100 cm^−1^ differences. For the (H)ZnNC systems as described by Molpro, the NC stretching frequency is about 19 cm^−1^ higher for the hydride, while for (H)ZnCN systems the difference is 21 cm^−1^. Gaussian values do not follow this pattern; see discussion below.

The 12–13 shift for the observed cyano stretch 2162.2–2114.9 is 47.3 cm^−1^, and it is less, 40.6, for the isocyanide. The observed 14/15 ratio for ZnNC is 1.01651 and the shift is 33.6 cm^−1^ while the computed shift for ZnCN is 32.9 cm^−1^ and the ratio is 1.01466. Thus the N moves more in the isocyanide than in the cyanide, which is obvious from the Zn−N−C structural formula.

The ^12^C/^13^C ratio from CCSD(T) for isocyano stretching frequencies in HZnNC, (2099.3/2059.1=1.01952), is characteristic of an isocyano stretch. The CCSD(T) band at 2028.2 cm^−1^ is unaffected by ^13^C isotopic substitution, which tells us that it is an almost pure Zn−H stretching mode. This is borne out by the computed ^1^H/^2^H isotopic ratio, (2028.2/1446.7)=1.40195. Note that CCSD(T) estimates the isocyano stretch very accurately in ZnNC, (2075 cm^−1^ observed, 2083 cm^−1^ calculated) but is less faithful to the hydride molecule's H−Zn stretch (2098 cm^−1^ for this ionic bond, 2074 cm^−1^).

Comparisons among computed stretching frequencies are possible. The significance of the perturbative triples (PT) in the CCSD(T) correction is minor for the Zn−H stretch, and for the cyano‐ and isocyano hydrides; it adds +2.8 cm^−1^ and +0.8 cm^−1^ to the cyano and isocyano H−Zn modes respectively. These modes, at 2010.8 and 2028.2 cm^−1^, are almost entirely local, and lie well to the blue of the asymmetric stretch for ZnH_2_ at 1870.2 cm^−1^. These ionic bonds are more difficult to model. The cyanide and isocyanide Zn−C and Zn−N stretches in the hydrides are virtually unaffected by the PT as well.

However the PT correction softens the cyano and isocyano stretches in the hydrogen compounds, by −70.5 and −63.3 cm^−1^. For the Zn(C,N) radicals, the PT correction softens the isocyano stretch by about that same amount (−64.6 cm^−1^), but stiffens the cyano stretch slightly (+17.3 cm^−1^). This anomaly, suggesting a flaw in the PT for the ZnCN, will be encountered in the CdCN and HgCN radical analogs as well.

## 3 Cadmium Studies

### 3.1 Spectroscopic Results and Discussion (Cadmium Systems)

**Figure 4:**
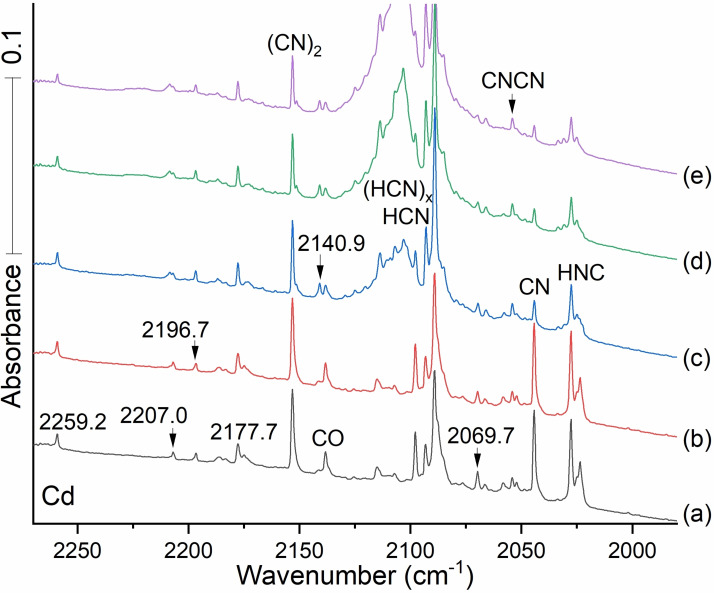
Infrared spectra between 2270 and 1950 cm^−1^ for the reaction products from laser ablated Cd co‐deposited with 0.2 % HCN in argon. Spectrum (a) grey: after laser ablated Cd co‐deposited with Ar/HCN during deposition for 120 min. Spectrum (b) red: after annealing to 10 K and cooling back to 5 K, Spectrum (c) blue: after annealing to 20 K and cooling back to 5 K. Spectrum (d) green: after annealing to 25 K and cooling back to 5 K and (e) purple: after annealing to 30 K and cooling back to 5 K. CO (2138.4 cm^−1^) is a very strong absorber, and a trace impurity in our vacuum system. ^13^CO is made from H^13^CN in the ablation process. *Notice the growth of both CN and (CN)_2_ on annealing to 10 K, which suggests that C and N atoms are also produced by the ablation process. The HNC precursor isomer also increases owing to the diffusion and reaction of H atoms with the CN radical*.

Figure 4 displays infrared spectra for cadmium atoms condensed in argon with 0.2 % HCN. Familiar nonmetal species including CO, CN, HCN, HNC, cyanogen, and its CNCN isomer, are identified explicitly. Upon first deposition (spectrum a) features appear at 2259.2, 2207.0, 2177.7, and 2069.7 cm^−1^. These signals persist through first annealing (spectrum b). Upon first annealing a new band emerges at 2196.7 cm^−1^, and upon annealing to 20 K. (spectrum c) another band appears at 2140.9 cm^−1^.

#### 3.1.1 CdCN and CdNC

The spectra of cadmium cyanides and isocyanides are slightly lower in frequency than those observed for the zinc cyanides and isocyanides. The isocyano stretching mode for CdNC is observed at 2069.7 cm^−1^ in the cyanogen reaction.^[19]^ The band at 2140.9 cm^−1^ is a candidate for the cyano stretch for CdCN, which is 21.3 lower than the analogous mode in behavior for ZnCN, and the CdNC product is only 4.9 cm^−1^ lower than its zinc analog.

These and other comparisons suggest that Cd acts like a heavier isotope of Zn. Although the Cd product bands are weaker, photolysis decreases the higher energy CdNC isomer in favor of the lower energy CdCN isomer.

#### 3.1.2 HCdCN and HCdNC

By analogy to observations in the Zn system, we expect to find the products of H radical‐CdCN combination. In Figure 4 we see new bands at 2177.7 and 2140.9 cm^−1^ separated by just 36.8 cm^−1^. In analogy to the Zinc case, these modes can reasonably be associated with the cyano stretches of the HCdCN and CdCN molecules. The bands observed at 1884.3 and 1864.8 cm^−1^ (see Figure 6) are candidates for Cd−H stretches in HCdNC and HCdCN. In this case the lower observed band at 2140.9 goes with the lower computed frequency at 2167 and the lower terminal Cd−H frequency at 1864.8 cm^−1^.

#### 3.1.3 Isotopic Effects

**Figure 5:**
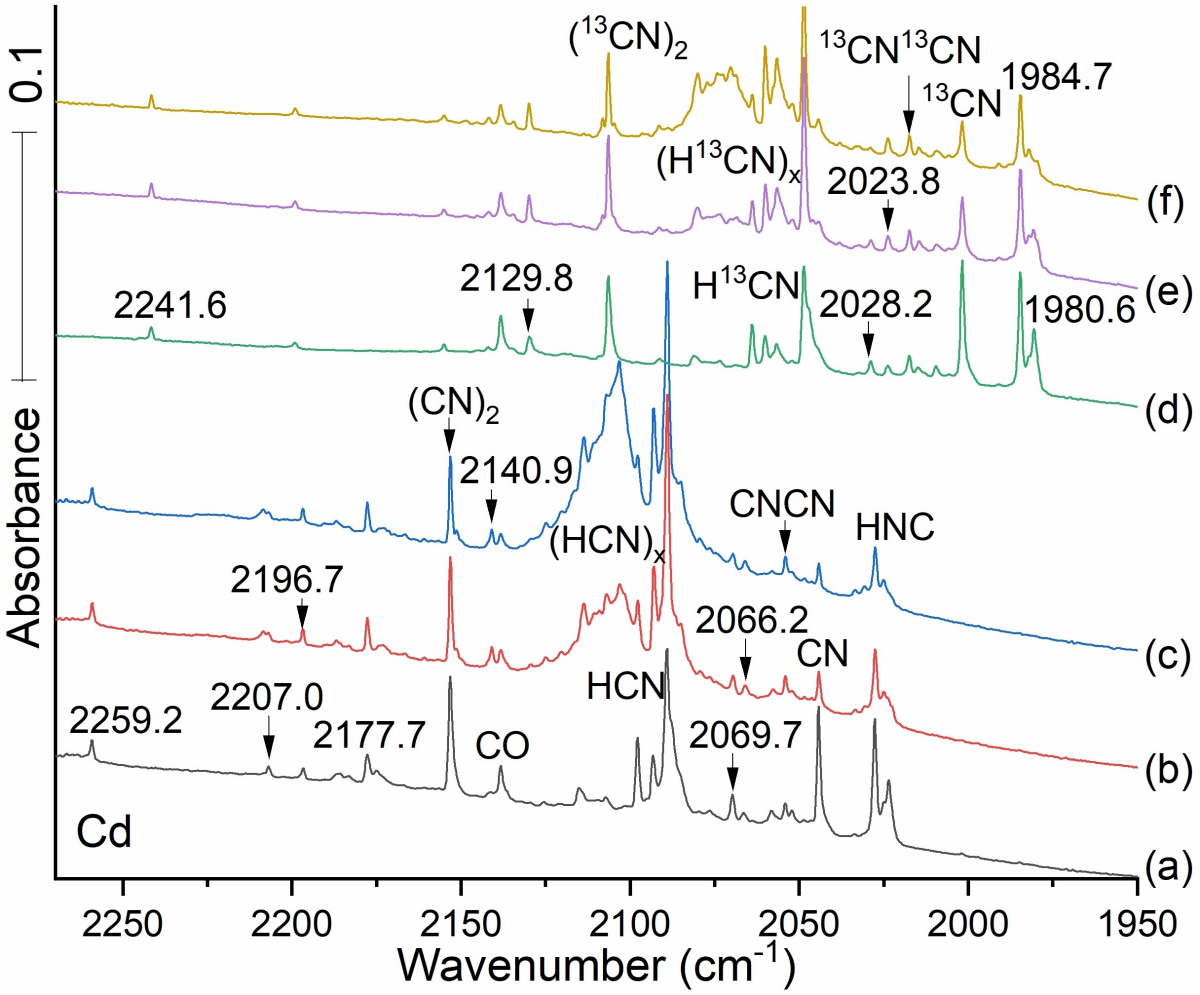
Infrared spectra between 2275 and 1950 cm^−1^ of the reaction products from laser ablated Cd co‐deposited with HCN. Spectra (a–c): Cd co‐deposited with 0.2 % H^12^CN. Spectra (d–f): Cd co‐deposited with 0.2 % H^13^CN (d–f) in argon at 5 K. Spectrum (a) gray and (d) green after sample co‐deposition for 120 min, Spectrum (b) red and (e) Fig 5, continued: purple after annealing to 15 K and cooling back to 5 K. Spectrum (c) blue and (f) brown after annealing to 20 K and cooling back to 5 K.

The strong isocyano absorption at 2069.7 cm^−1^ has a ^13^C counterpart at 2028.2 cm^−1^ (Figure 5, Table 3). Its ^12^C/^13^C isotopic frequency ratio, 1.02046, is only slightly higher than the 1.01996 value found for the zinc counterpart. We may infer a larger amplitude motion for C against the heavier metal in Cd−C−N. The ^12^C/^13^C frequency ratio for the 2177.7 cm^−1^ band is 1.02249, which suggests the cyano isomer HCdC−N and another C−N band. Figure 5(b) red shows a substantial 75 % decrease in the CN radical and an increase in HCN and cyanogen NCCN on annealing to 15 K, which also happens to the carbon‐13 analogs.

**Figure 6:**
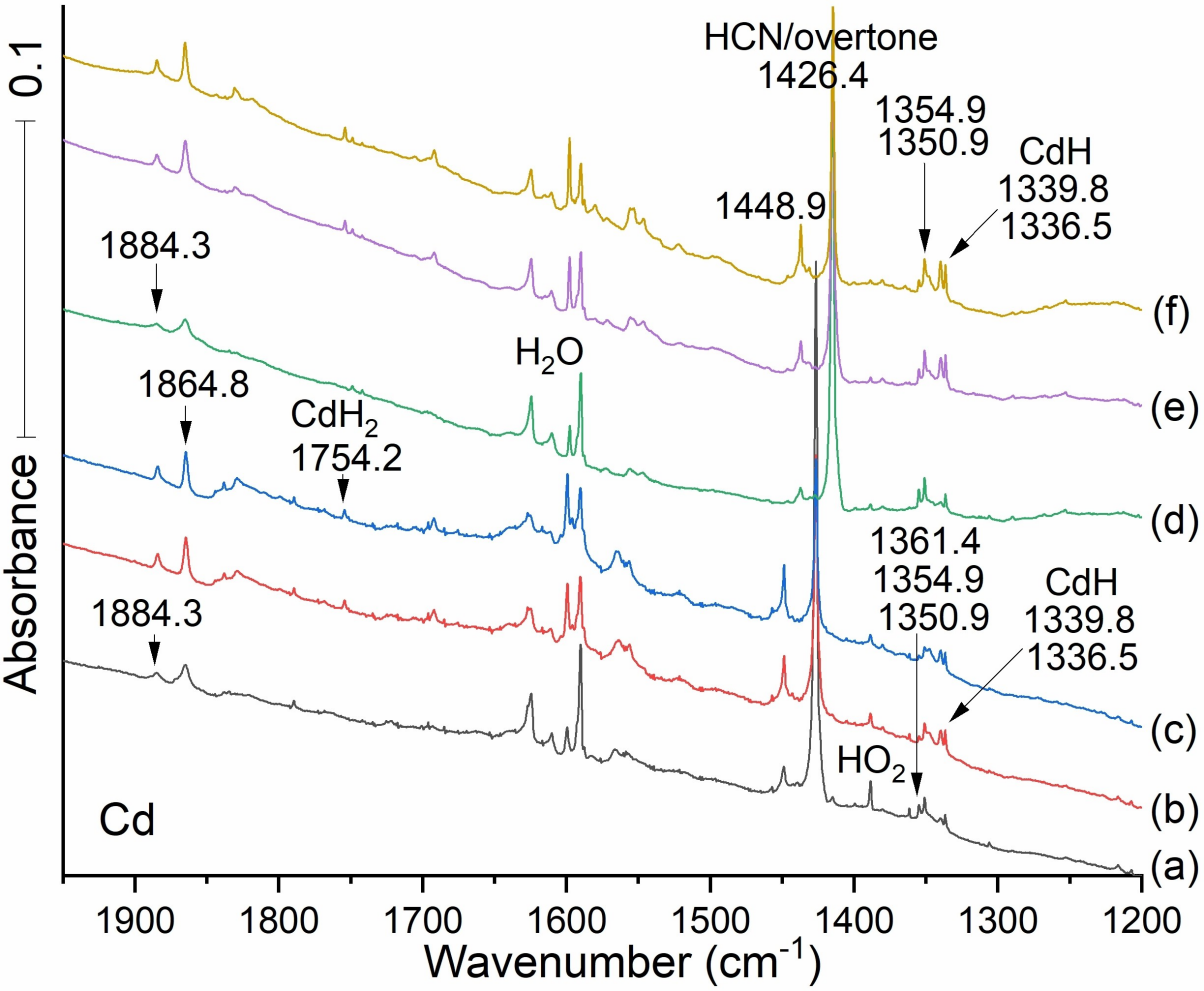
Infrared spectra between 1950 and 1200 cm^−1^ for the reaction products from laser ablated Cd co‐deposited with HCN. Spectra (a–c) Cd co‐deposited with 0.2 % (a–c) H^12^CN and spectra (d–f) Cd co‐deposited with 0.2 % H^13^CN (d–f) in argon at 5 K. Spectrum (a) grey and (d) green after sample co‐deposition for 120 min, Spectrum (b) red and (e) purple after annealing to 15 K and cooling back to 5 K. Spectrum (c) blue and (f) brown after annealing to 20 K and cooling back to 5 K.

**Table 3:** Frequencies [cm^−1^] observed from reactions of Cd atoms with H^12^CN and H^13^CN in excess argon at 5 K. Bold type marks the major trapping site. Harmonic frequencies computed by Molpro CCSD(T)/aug‐cc‐pVTZ guided the assignment of structures and vibrational modes.^[a]^

Cadmium				
^12^C	^13^C	Isotopic Ratio	Computed	Species
2177.7] (ia)	2129.8 (ia)	1.02249	2190	HCdCN
2140.9	Under (H^13^CN)_x_	–	2167	CdCN
2088 sh	Under (H^13^CN)_x_	–	2094	HCdNC
2069.7, 2066.2	2028.2, 2023.8	1.02046	2077	CdNC, site variants
1884.3 (da)	1884.3 (da)		1925	HCdNC Cd−H stretch
1864.8 (ia)	1864.8 (da)		1908	HCdCN Cd−H stretch
1754.2 {1756.2}	{1260.3	{1.39348}		CdH_2_ and CdD_2_ in Ar with a H_2_/D_2_ mix^6^
1339.8 {1339.4}	{974.4}	{1.37459}		CdH and CdD in Ar with a H_2_/D_2_ mix^6^
[1771.53]^[35]^	[1278.3]^[35]^	1.38585		CdH_2_ and CdD_2_ (gas)^[35]^

[a] Band behavior: ia=increase on annealing; da=decrease on annealing; dp=decrease on photolysis; sh=shoulder. Frequency values in curly brackets were observed using other methods.

The signal at 2140.9 cm^−1^ shown in Figure 5 spectrum c obtained after annealing to 20 K, can be attributed to CdCN by indirect argument. The signal for the cyano stretch for Cd^13^CN is obscured by the intense absorption of HCN, but (1) the proximity of the 2140.9 cm^−1^ band to the 2177.7 cm^−1^ band attributable to the HCd^12^CN cyano stretch and (2) HCd^13^CN's isotopic counterpart at 2129.8 cm^−1^ with ratio 1.02249, together support the CdCN assignment.

### 3.2 Computational Modeling (Cadmium Systems)

#### 3.2.1 Vibrational Frequencies for Isotopic Variants of Cadmium Systems

Table 4 contains computed vibrational frequencies for isotopic variants of the cadmium systems. As we found for the zinc systems, computed ^12^C/^13^C frequency ratios clearly distinguish cyano from isocyano systems. The ratios calculated from the CCSD and CCSD(T) frequencies for CdCN are 1.02255 and 1.02252, in close agreement with the zinc analogs. Unfortunately, the Cd^12^CN/Cd^13^CN frequency ratio cannot be measured because the Cd^13^CN frequency is obscured by the H ^12^CN band present as a 1 % impurity in the H^13^CN sample. The observed ^12^C/^13^C frequency ratio for CdNC is 1.02046 while the computed values are 1.02026 and 1.02023 using CCSD and CCSD(T), respectively.

**Table 4:** Calculations for cadmium cyanide and isocyanide and their hydride isotopic frequencies [cm^−1^] and intensities [km/mol]. From Gaussian calculations with the aug‐cc‐pVTZ‐pp basis.

CCSD/aug‐cc‐pVTZ/aug‐cc‐pVTZ‐pp					
Species	ν1 σ (C−N) (Intensity)	(^12^C/^13^C)^a^ (^14^N/^15^N) Isotopic Freq Ratios	ν2 σ (Intensity)	ν4 σ (Intensity)	ν1–ν4
CdCN	2253.7 (21)	cal	369.2 (56)		
Cd^13^CN	2204.0 (17)	1.02255	364.0 (54)		
CdC^15^N	2221.0 (22)	1.01472	363.3 (54)		
CdNC	2144.7 (258)		397.3 (78)		
CdN^13^C	2102.1 (258)	1.02026	390.8 (75)		
Cd^15^NC	2109.6 (241)	1.01664	391.6 (76)		
HCdCN	2261.2 (18)		411.9 (38)	1911.0 (98)	351.2
DCdCN	2261.2 (18)		411.5 (37)	1357.8 (52)	
HCd^13^CN	2210.8 (16)	1.02280	406.1 (37)	1911.0 (98)	
HCdC^15^N	2228.8 (19)	1.01454	405.1 (37)	1911.0 (98)	
HCdNC	2160.0 (249)		450.0 (55)	1925.3 (70)	234.7
DCdNC	2160.0 (247)		449.5 (55)	1368.3 (40)	
HCdN^13^C	2117.8 (249)	1.01993	442.5 (53)	1925.3 (67)	
HCd^15^NC	2124.1 (234)	1.01690	443.7 (54)	1925.3 (70)

CCSD(T)/aug‐cc‐pVTZ/aug‐cc‐pVTZ‐pp					
Species	ν1 σ	(^12^C/^13^C) or (^14^N/^15^N) Isotopic Ratios	ν2 σ	ν4 σ	ν1–ν4
CdCN	2251.9		358.0		
Cd^13^CN	2202.3	1.02252	352.9		
CdC^15^N	2219.1	1.01478	352.2		
CdNC	2078.1		392.8		
CdN^13^C	2036.9	1.02023	386.4		
Cd^15^NC	2044.0	1.01668	387.3		
HCdCN	2189.4		409.6	1907.5	281.9
DCdCN	2189.4		409.2	1355.4	
HCd^13^CN	2140.5	1.02284	403.9	1907.5	
HCdC^15^N	2158.2	1.01446	402.9	1907.5	
HCdNC	2098.7		451.7	1949.7	149.0
DCdNC	2098.7		453.1	1385.5	
HCdN^13^C	2057.9	1.01983	446.0	1949.6	
HCd^15^NC	2063.7	1.01696	447.3	1949.6

The ratios of CN stretching frequencies for CdC^14^N/CdC^15^N isotopic substitution are 1.01472 according to CCSD calculation, 1.01478 by CCSD(T). The ^14^N/^15^N frequency ratio for CdNC is 1.01664 according to CCSD and 1.01668 by CCSD(T). Comparison of the CdCN ratios suggests that there is more N motion and less C movement in the isocyanide than in the cyanide.

The parallel between (C and N) stretching frequency isotope ratios in the Cd(C,N) and HCd(C,N) species is plain. For HCdCN, the CCSD and CCSD(T) values for the ^12^C/^13^C frequency ratios are 1.02280 and 1.02284 respectively, very close to the ratios for CdCN. The corresponding computed ^12^C/^13^C values for HCdNC are 1.01993 and 1.01983. The ^14^N/^15^N frequency ratios for HCdNC are 1.01690 according to CCSD and 1.01696 by CCSD(T), in close agreement with corresponding values for CdNC.

Calculations further show that the new band observed at 1864.8 cm^−1^ is due to the Cd−H stretching mode of HCdCN (computed at 1907.5 cm^−1^), and the higher new band in this region at 1884.3 cm^−1^ is due to the Cd−H stretching mode for the HCdNC isomer (computed at 1949.6 cm^−1^). CCSD and CCSD(T) calculations agree closely in ^1^H/^2^H isotopic ratio for the CdH stretch (1.40742 and 1.49733 respectively), but both overestimate the H−Cd stretch value considerably.

### 3.3 Discussion of (H)Cd−CN, Cd−H and (C−N) stretching frequencies

As in the HZn(C,N) systems, the significance of the perturbative triples (PT) in the CCSD(T) correction is modest for the H−Cd cyano (−3.5 cm^−1^) and isocyano (24.4 cm^−1^) stretches. The PT corrections are −9.2 cm^−1^ and −4.5 cm^−1^ for the Cd−C and Cd−N stretches in the two isomers. However the PT correction softens the cyano and isocyano stretches in the hydrogen compounds, by −70.8 cm^−1^ and −61.3 cm^−1^ respectively. For the Cd(C,N) radicals, the PT correction softens the isocyano stretch by a like amount (−66.6 cm^−1^), but has little effect on the cyano stretch (−1.8 cm^−1^). This anomaly, seen in more striking form in the Zn system, will be encountered in the Hg systems as well.

There are some other persistent features of the stretching frequency differences between cyano and isocyano systems. Adding H stiffens the (H)Cd−(C=N) frequencies slightly, and the C=N frequencies for both radicals and hydrogenated compounds are higher for the cyano systems than for the isocyano systems, by a like amount. Once again the PT term for the CdCN system is anomalous. The H−Cd stretch is stiffer for the isocyano system, and there is a noticeable effect of the PT correction.

## 4 Mercury Studies

### 4.1 Spectroscopic Results and Discussion (Mercury Systems)

**Figure 7:**
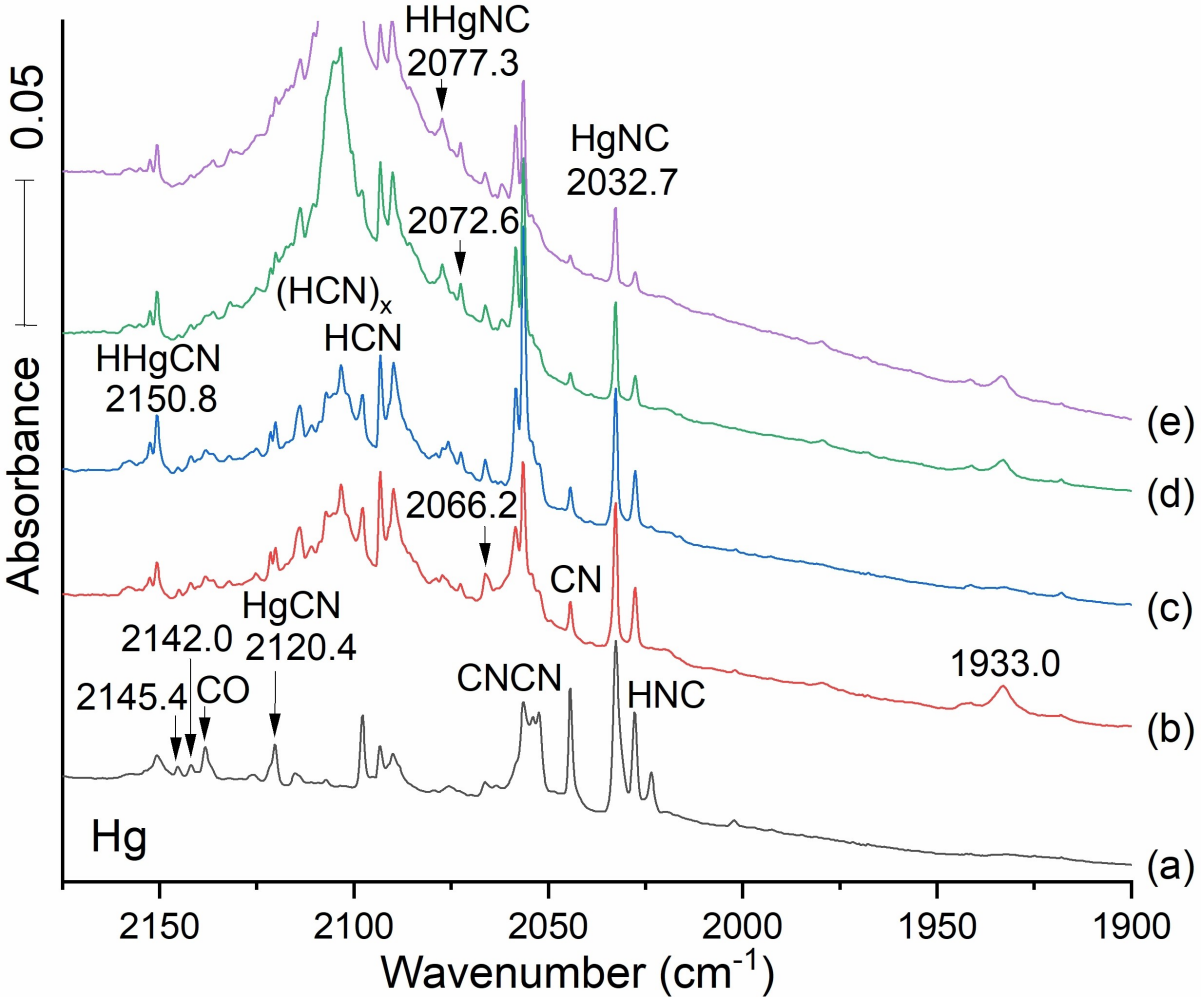
Infrared spectra of the reaction products of laser ablated Hg from a dental amalgam target with 0.2 % HCN in argon during condensation at 5 K. The sample was exposed to ablation plume Hg resonance radiation during the deposition process. The bottom spectrum (a) grey followed co‐deposition of the reagents for 120 min spectrum, (b) red recorded after annealing to 20 K and cooling back to 5 K. Spectrum (c) blue taken after full mercury arc photolysis for 20 min. Spectrum (d) green followed annealing to 30 K and spectrum (e) purple followed annealing to 35 K and cooling back to 5 K.

We generated Hg atoms by laser ablation of a 50 % Hg dental amalgam target, a new procedure developed in our labs.[^9^, ^17^] A picture of this target is shown in supporting Information P‐1. The Volatile Hg is produced preferentially, leaving behind the other more refractory components of the amalgam. Co‐deposition at 5 K with 0.2 % HCN and argon followed by further processing produced the spectra displayed in Figure 7.

#### 4.1.1 Appearance of HgCN, HgNC, HHgCN and HHgNC

Besides the signals associated with familiar non‐metallic species, the first deposition (spectrum a) produced bands at 2032.7 and 2120.4 cm^−1^ as well as 2150.8 cm^−1^ and 2066.2 cm^−1^. The first annealing produced a new absorption (spectrum b, red) at 1933.0 cm^−1^ and had little effect on the other bands: All bands were enhanced on photolysis (spectrum c) except the 1933 cm^−1^ absorption, which was erased (spectrum c, blue). This feature will be considered after more experiments and calculations are done on possibilities for the 1933 band.

We will be following analogies set forth in the above Zn and Cd studies, which lead us to anticipate an intense signal from the HgNC isocyano stretch labeled in the spectra of the Figure 7 deposited sample. The strong band at 2032.7 cm^−1^ (37 cm^−1^ below CdNC), is a good candidate. The feature at 2120.4 cm^−1^ meets our expectation for an HgCN stretch at ca. 100 cm^−1^ higher frequency and less intensity than the isocyano stretch. Even so we expect that there are more of the weaker absorbing more stable HgCN molecules in our matrix: Band intensity is central to this work.

#### 4.1.2 Isotopic Effects

**Figure 8:**
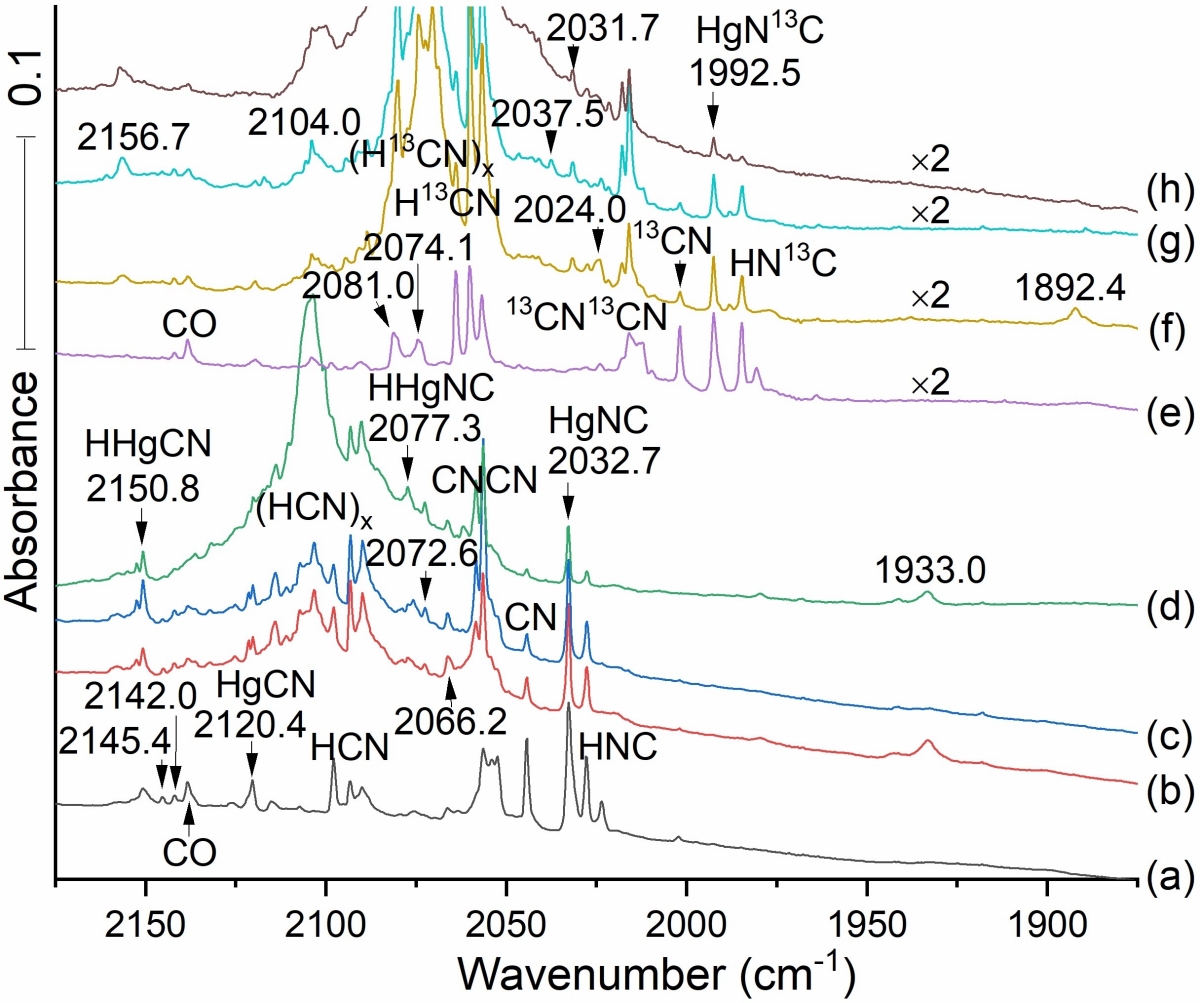
Infrared spectra of the products of laser ablated Hg atom reactions from a dental amalgam target. Spectra (a–d), Hg with 0.2 % H^12^CN in argon: spectra (e–h), Hg with 0.2 % H^13^CN in argon. Spectra (a) and (e) are observed after co‐deposition for 2 h. Spectra (b) and (f) (red, brown) recorded after annealing to 20 K and cooling back to 5 K. Spectra (c) and (g) (blue) recorded after 20 min irradiation with full medium pressure mercury arc lamp. Spectra (d), green, and (h) grey are recorded after annealing to 30 K and cooling back to5 K.

These proposals are borne out by the presence of transitions at 1992.5 cm^−1^ and 2074.1 cm^−1^ in the spectra produced with H^13^CN (Figure 8). These are linked to primary product absorptions for HgNC and HgCN, respectively to be assigned here. The isotopic ratio in the first case is 1.02018 (Table 5), characteristic of the isocyano stretch, and in the second case is 1.02232, close to the value for the cyano stretch established here in Zn and Cd systems. These ^12^C/^13^C red shifts by 46.3 and 40.2 cm^−1^ to lower frequencies follow the profiles for Cd and Zn (compare Tables 1 and 2 for Zn, 3 and 4 for Cd above).

**Table 5:** Observed frequencies and isotopic ratios for species produced by codeposition of Hg and HCN with argon (A) and reported frequencies for hydride HgH_2_ (B).^[f]^

MERCURY SYSTEMS A: cyanides and isocyanides			
^12^C	^13^C	Isotopic Ratio	Species
2150.8	May be hidden by (H^13^CN)_x_	–	HHgCN
2120.4	2074.1	1.02232	HgCN &HNC
(2066.2)	2114.9	102236	(HHgN−C)
2027.6			HN−C
2032.7 (da)	1992.5 (da)	1.02018	HgN−C
1933.0 (ia, dp)	1892.4(ia,dp)	1.02145	Work in progress

MERCURY SYSTEMS B: hydrides			
1912.8 (gas)			HgH_2_ asym str (a)
1895.3 (Argon mat.)			HgH_2_ asym str (b)
1900.4: 1910.3 (e)			
1209 (H_2_/D_2_)	899.8, 909.7	1.34363	Hg−H str (c)
Tab5 cont 1203.2 (gas)	896.1	1.34348	Hg−H str (d)
1205			Hg−H str

[a] Ref. [37]. [b] Ref. [38]. [c] Ref. [8]. [d] Ref. [39]. [e] Ref. [40]. [f]  Band behavior: (ia=increase on annealing, da=decrease on annealing),dp=decrease on photolysis.

The information that leads to assignment of infrared bands for HgCN, HgNC, HHgCN and HHgNC is presented here in Table 5.

We anticipate signals for CN stretches in HHgCN and HHgNC that increase on annealing: Our Molpro calculation with CCSD(T) (Table 6) predicts the HHgCN cyano stretching mode to lie 33 cm^−1^ higher than the HgCN's CN stretch. The weak band observed at 2066.2 is 33 cm^−1^ higher than the HgNC band at 2032.7 cm^−1^: It does not grow substantially on annealing, but it is in the region where it could be covered by reforming HCN. Thus the feature at 2066.2 cm^−1^ may be attributed to HHgNC, but we cannot be as confident in such an assignment as we are with the isocyanide isomer. On the other hand the 2150.8 band does grow on first annealing and photolysis and it is probably due to HHgNC. This cyanide at 2150.8 and isocyanide at 2066.2 differ by 84.6 cm^−1^ which is almost the ca. 100 cm^−1^ expected difference.

**Table 6:** Calculations on HgH_2_

Model (Harmonic)	Sym. Stretch	Asym. Stretch	Hg−H distance
CCSD/aug…pp	2110.6	1986.9 obs. 1895	1.6361
CCSD(T)/aug…pp	2098.6	1979.0	1.6366

### 4.2 Computational Modeling (Mercury Systems)

#### 4.2.1 Vibrational Frequencies for Isotopic Variants of the Hg systems

CCSD/aug‐cc‐pVTZ/aug‐cc‐pVTZ‐pp and CCSD(T)/aug‐cc‐pVTZ/aug‐cc‐pVTZ‐pp calculations of selected frequencies are collected in Table 6. Following the pattern for Zn and Cd, the computed cyanide stretching frequencies in HgNC and HHgNC are about 100 cm^−1^ higher than the corresponding values in the isocyanides. Likewise the ^12^C/^13^C isotopic ratios are larger for the cyanides than for the isocyanides. The Hg−H stretches are placed at 2190.5 by CCSD and 2175.7 by CCSD(T) for HHgCN and at 2255.6 by CCSD and 2243.9 cm^−1^ by CCSD(T) for HHgNC. They are identified by calculated ^1^H/^2^H isotopic ratios 1.40986 and 1.41072. However these computed frequency values are higher than those assigned to Hg−H stretches in the experimental spectra (Table 7).

**Table 7:** Calculations for stretching modes of mercury cyanide and isocyanide and their hydride isotopic frequencies [cm^−1^] and intensities [km/mol].

CCSD/aug‐cc‐pVTZ (pp)				
Species	ν1 σ (Intensity)	Isotopic Fr ratios^[a]^	ν2 σ (Intensity)	ν3 σ (Intensity)
HgCN	2265.1 (11)		351.1 (30)	
Hg^13^CN	2215.1 (9)	1.02257	345.6 (29)	
HgC^15^N	2232.2 (12)	1.01474	345.0 (29)	
HgNC	2140.0 (252)		376.8 (42)	
HgN^13^C	2097.5 (250)	1.02026	370.1 (40)	
Hg^15^NC	2104.8 (238)	1.01672	370.9 (41)	
HHgCN	2278.2 (9)		432.5 (35)	2190.5 (93)
DHgCN	2277.3 (16)		432.5 (34)	1553.7 (45)
HHg^13^CN	2228.1 (2)	1.02248	426.0 (34)	2189.3 (93)
HHgC^15^N	2246.8 (6)	1.01443	424.8 (33)	2189.8 (97)
HHgNC	2167.7 (294))		463.8 (57)	2255.6 (32)
DHgNC	2168.3 (275)		463.7 (57)	1598.9 (28)
HHgN^13^C	2125.9 (288)	1.01966	455.3 (54)	2255.4 (37)
HHg^15^NC	2131.4 (272)	1.01703	456.8 (55)	2255.4 (38)

CCSD(T)/aug‐cc‐pVTZ/aug‐cc‐pVTZ‐pp				
Species	ν1 σ	Isotopic Fr Ratios^a^	ν2 σ	ν4 σ
HgCN	2266.5		346.8	
Hg^13^CN	2216.6	1.02251	341.5	
HgC^15^N	2233.5	1.01478	340.8	
HgNC	2072.1		366.1	
HgN^13^C	2031.1	1.02019	359.6	
Hg^15^NC	2044.0	1.01375	387.3	
HHgCN	2207.3		429.7	2175.7
DHgCN	2203.5		429.7	1545.2
HHg^13^CN	2182.9	1.01118	423.3	2150.3
HHgC^15^N	2187.3	1.00914	422.0	2164.8
HHgNC	2100.9		459.7	2243.9
DHgNC	2101.4		459.6	1590.7
HHgN^13^C	2060.6	1.01956	451.3	2243.8
HHg^15^NC	2065.5	1.01714	452.8	2243.8

[a] (^12^C/^13^C) or (^14^N/^15^N).

#### 4.2.2 Discussion of (H)Hg−CN, Hg−H and (C, N) stretching frequencies

The significance of the perturbative triples (PT) correction in CCSD(T) is minor in the Hg−H stretches, −14.8 cm^−1^ for the cyano isomer and −11.7 cm^−1^ for the isocyano isomer. The shift is only −2.8 cm^−1^ for the Hg to C (cyano) stretch and −4.2 cm^−1^ for the Hg to N (isocyano) stretch.

However the PT correction softens (red‐shifts) the cyano and isocyano stretches in the hydrides. For the cyanide softening is −70.9 cm^−1^, while for the isocyanide the shift is −79.6 cm^−1^. For the Hg(C,N) radicals, the PT correction softens the isocyano stretch by −67.9 cm^−1^, but has little effect on the cyano stretch, the shift being +1.4 cm^−1^. However these computed values are far higher than those observed here. This anomaly is seen throughout the metals. The most immediately relevant impact is on the frequency assignment of the CN stretches in the MCN species, which is discussed below. There are some other persistent features of the stretching frequency differences between cyano and isocyano systems. Adding H increases the (H)Hg to cyano or isocyano stretching frequencies slightly, and the frequencies for both radicals and hydrogenated compounds are higher for the cyano systems than for the isocyano systems, by a like amount. The Hg−H stretching frequency is higher for the isocyano system.

## 5 Comparisons within the Group 12 Family

A good way to celebrate the chemistry of the Group 12 metals is to make comparisons within this family of metals. In the following sections we describe bonding in the Zn, Cd, and Hg cyanides and isocyanides and their mono hydrides, and draw some generalizations on the relative energies, the geometries, and the vibrational spectra of compounds of Zn, Cd, and Hg.

### 5.1 Bonding in (H)HgNC

The Zn, Cd, and Hg cyanides and isocyanides are qualitatively similar in bonding. Starting with the MOs for N_2_, we find symmetry‐breaking polarization produces clear correlates in the CN anion to the central sigma bond, two pi bonds, and two lone pairs of N_2_. As a metal cation with one odd *ns* electron approaches along the sigma axis, a sigma bond forms to that metal. The ground state of the triatomic is ^2^Σ.^[23]^

**Figure 9:**
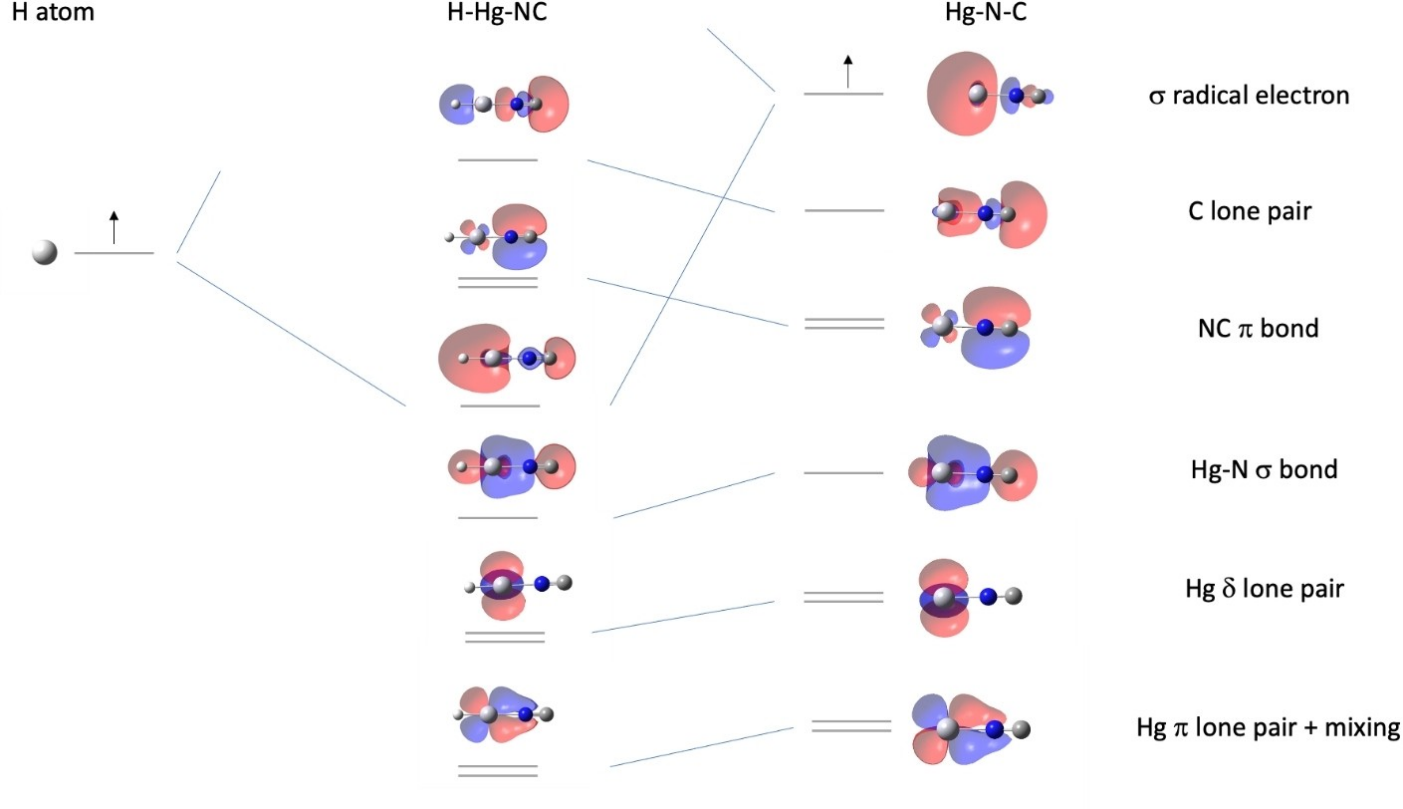
Partial bonding diagram for HgNC and HHgNC. In HgNC the singly occupied MO is primarily the Hg sigma 6 s AO. The Hg delta lone pairs are isolated (d_2_, d_−2_) AOs, while the Hg (d_1_, d_−1_) pi lone pairs mix with carbon's (p_1_, p_−1_) AOs. These lone pairs lie lower than the Hg−N σ bond and the NC π bonds. In the formation of HHgNC, the new sigma H−Hg bond is a mixture of the HgNC singly occupied 6 s AO and the H 1s AO. The pairing of odd electrons makes the process strongly exothermic and leads to closed shell systems.

The qualitative nature of bonding in the Zn, Cd, and Hg hydrides is also very similar for all species. Figure 9 shows the specific case of (H)HgNC. The odd electron on HgNC found on the Hg atom (mainly the 6s AO) pairs with the odd electron of H atom as they approach one another along the molecular axis. The HOMO of the hydride is primarily of the C lone pair, with some destabilizing admixture of the sigma bond.

### 5.2 Relative Energies of Cyano and Isocyano Systems

**Figure 10:**
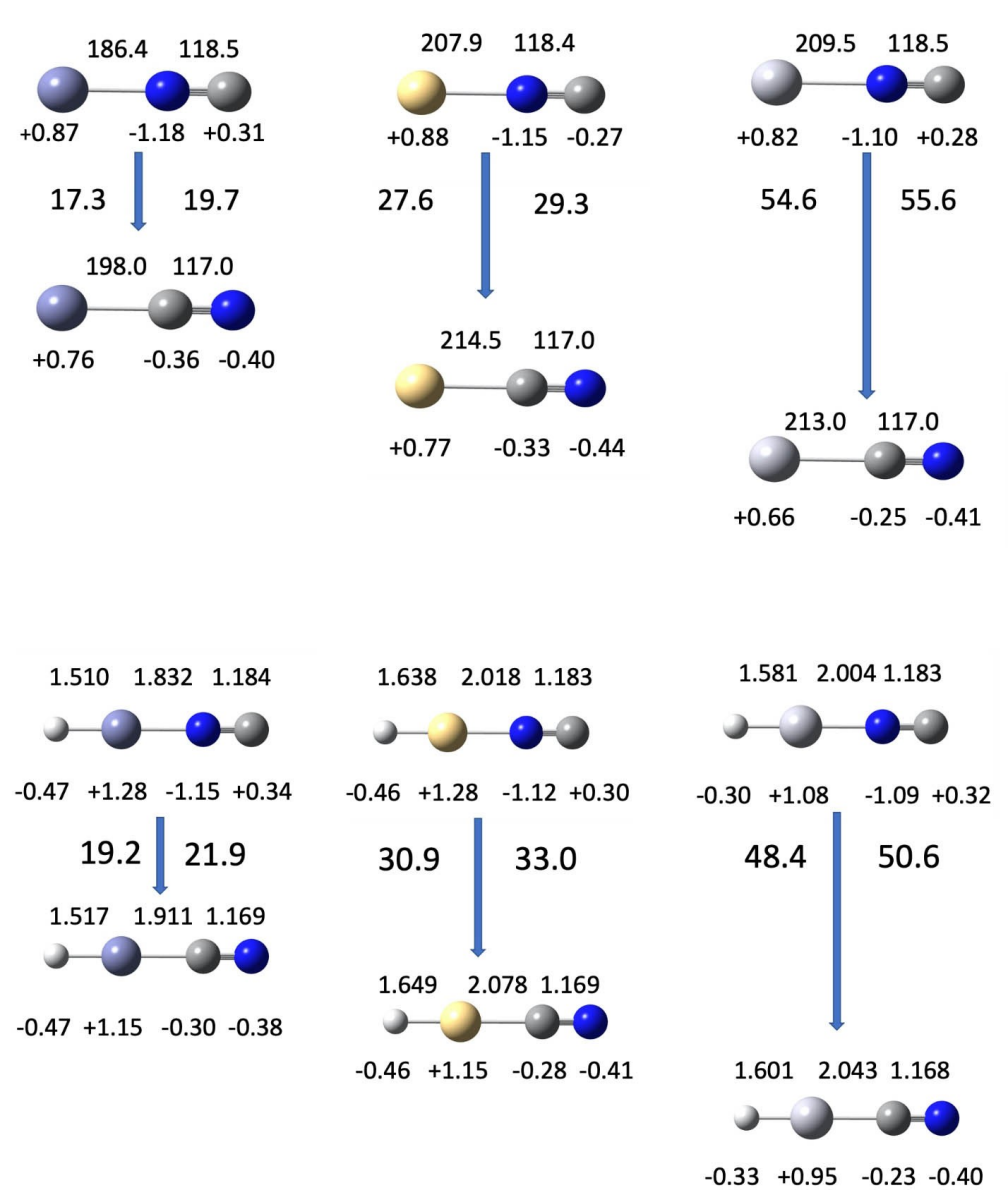
Relative energies, bond lengths, and natural charges from CCSD(T)/aug‐cc‐pVTZ (Zn) and aug‐cc‐pVTZ‐pp (Cd, Hg). Relative energy is in kJ/mol; CCSD values appear left of each arrow, CCSD(T) at right. Bond lengths appear above each molecular image, while natural charges appear below each atom.

The data collected below in Table 8 and pictured in Figure 10 show that the cyano species are consistently more stable than isocyano species, with the difference increasing down Group 12. CCSD and CCSD(T) estimates (kJ/mol) are consistent within about 5 kJ/mol.

**Table 8:** Relative stability of cyanides and isocyanides: E(isocyano) – E(cyano) [kJ/mol]. Energies obtained with CCSD(T)/aug‐cc‐pVTZ (Zn) and aug‐cc‐pVTZ‐pp (Cd and Hg)

ΔE (Isocyano‐Cyano)	ZnNC−ZnCN	HZnNC−HZnCN
CCSD	17.3	19.2
CCSD(T)	19.7	21.9
Species	CdNC−CdCN	HCdNC−HCdCN
CCSD	27.4	30.9
CCSD(T)	29.3	33.0
Species	HgNC−HgCN	HHgNC−HHgCN
CCSD	54.6	48.4
CCSD(T)	55.6	50.6

Figure 10 shows bond lengths and natural charges for the triatomic Zn, Cd, and Hg cyanides and isocyanides and for the tetra‐atomic hydride cyanides. Parameters were obtained with CCSD(T)/aug‐cc‐pVTZ (Zn) and aug‐cc‐pVTZ‐pp (Cd and Hg). Energies are reported in both CCSD and CCSD(T), which agree closely, within 3 kJ/mol.

### 5.3 Geometry from CCSD and CCSD(T)

Table 8 contains calculated geometric parameters for the M−C−N cyanides and the M−N−C isocyanides. Note the persistently longer polar C−N distances in the metal isocyanides, and the relatively uniform charges on C and N atoms in the metal cyanides. Therefore the isocyanides present stronger infrared absorptions. This is also seen in the tetra‐atomic hydrides.

In every case the (H)M−CN metal to cyano carbon bond for cyano is longer for the (H)M−NC metal to nitrogen bond for isocyano systems. This may be a consequence of the smaller atomic radius for N compared with C, or greater inhomogeneity in charge in the isocyano structure compared with the cyano structure. Note, an accompanying shortening of the cyano bond relative to the isocyano bond is observed. Alternatively, this shortening may be a consequence of increasing electron density along the internuclear axis as the M−H bond is formed from the unpaired electrons on H and M. Adding H to form HMCN and HMNC has almost no effect on the C−N and N−C distances, but a shortening effect on the MC and MN bonds.

Microwave spectroscopic investigations of a mixtures of zinc vapor and cyanogen gas in a DC discharge observed the same two molecules ZnCN^[23]^ and HZnCN^[24]^ that we find with matrix isolation IR and laser ablated Zn atoms reacting with HCN in excess argon.

### 5.4 Trends in IR Spectra

Several features of the observed and computed IR spectra can be recognized.

1. **Greater intensity of isocyano stretch relative to cyano stretch**: Isocyanides show persistently (almost tenfold) higher isocyano stretching absorption intensities (CCSD computed) over the cyanides. The high intensities for the isocyano stretching modes are due to the more substantially nonuniform polar charge distribution in the (H)MNC N to C bond relative to that of the C to N bond in cyanides (see natural charges in Figure 10).
2. Isotopic frequency ratios distinguish cyano from isocyano species, and provide clues to the amplitudes of motion of C in MCN and N in MNC. The observed ^12^C/^13^C isotopic frequency ratios provide a reliable predictor of cyano and isocyano structures, and allow distinction of radical and closed shell systems. Broadly, cyano systems display ratios near 1.0210 or greater, while isocyano systems have smaller ratios. CCSD and CCSD(T) computations provide generally reliable estimates of isotopic frequency ratios, consistent with this behavior. The ultimate comparison for C atom movement in a vibration is to compare the ^12/13^C frequency ratios between the vibrations in ^12^C−^16^O (1.02252, Table 1 observed ratio for CO in solid argon) with the ratios for ^12^C/^13^C in the antisymmetric stretching modes for ^16^O^12^C−^16^O and ^16^O^13^C^16^O: Figure S5 shows the featured antisymmetric vibration in OCO spectra which are *matrix site split into two bands* so we have two values for the carbon dioxide *υ3* vibration, where the frequencies are given in Figure S5. The 12/13 ratios are 2339.2/2273.6=1.02885 and 2345.1/2279.5=1.02878: These numbers should be the same, but there are very small differences in the anharmonicity in the two isotopomers we are comparing. We round to four decimal places and get 1.0288 which is just an inkling larger than the 1.0225 CO value given above. So the C atom moves more in the antisymmetric mode in OCO and in M−C−N by comparison than it does in C−N just as it moves more in OCO than in CO. Thus, the special character of the really M−N−C antisymmetric N stretching mode in the isocyanide sustains more N movement than in the MN−C vibration of the cyanide.
3. **Shifts in isocyano stretching frequencies can be rationalized**: The observed N−C stretching frequencies for MCN's are similar for the three metals (range 2162–2120 cm^−1^) as are MNC isocyano stretching frequencies (range 2075–2033). The isocyano stretching frequency for CdNC (2069.8 cm^−1^) is just 4.8 cm^−1^ less than that of ZnNC (2074.6 cm^−1^), which may be attributed to coupling with the heavier Cd metal and an increase in the motion's reduced mass. The observed HgN−C frequency (2032.7 cm^−1^) is shifted by a greater amount, 37.1 cm^−1^, toward the red. This may be due to the greater relativistic effect for Hg (as modeled by the corrected pseudopotential and the basis aug‐cc‐pVTZ‐pp ^[33]^) leading to stronger Hg−N interaction and a still greater reduced mass for this asymmetric N motion between Hg and C. The pattern is echoed in the assigned isocyano HMNC frequencies, 2097.8 for M=Zn, a small red shift to 2088.0 for Cd, and a more substantial shift to 2066.2 cm^−1^ for Hg.
4. **CCSD(T) calculations are useful but must be evaluated with care**: CCSD(T) calculations with an extensive basis (specifically aug‐cc‐pVTZ for Zn with a small pseudopotential(pp) for Cd and Hg) are useful in assigning features of vibrational spectra for H−M cyanides and isocyanides. Relativistic effects, which would be significant for Hg and perhaps also for Cd, are represented in calculations only by the pseudopotential in the basis aug‐cc‐pVTZ‐pp. Such calculations fall short of best results for certain kinds of vibrations. M−H frequencies are overestimated in both CCSD and in CCSD(T). Cyano stretches for MCN radicals are sometimes overestimated, (see discussion below) while isocyano counterparts are accurately represented. HMCN and HMNC vibrations are well‐described, more accurately described.
5. **A correlation diagram permits easy evaluation of vibrational assignments**: The correlation between computed and assigned frequencies is shown in Figure 11
  **Figure 11:**
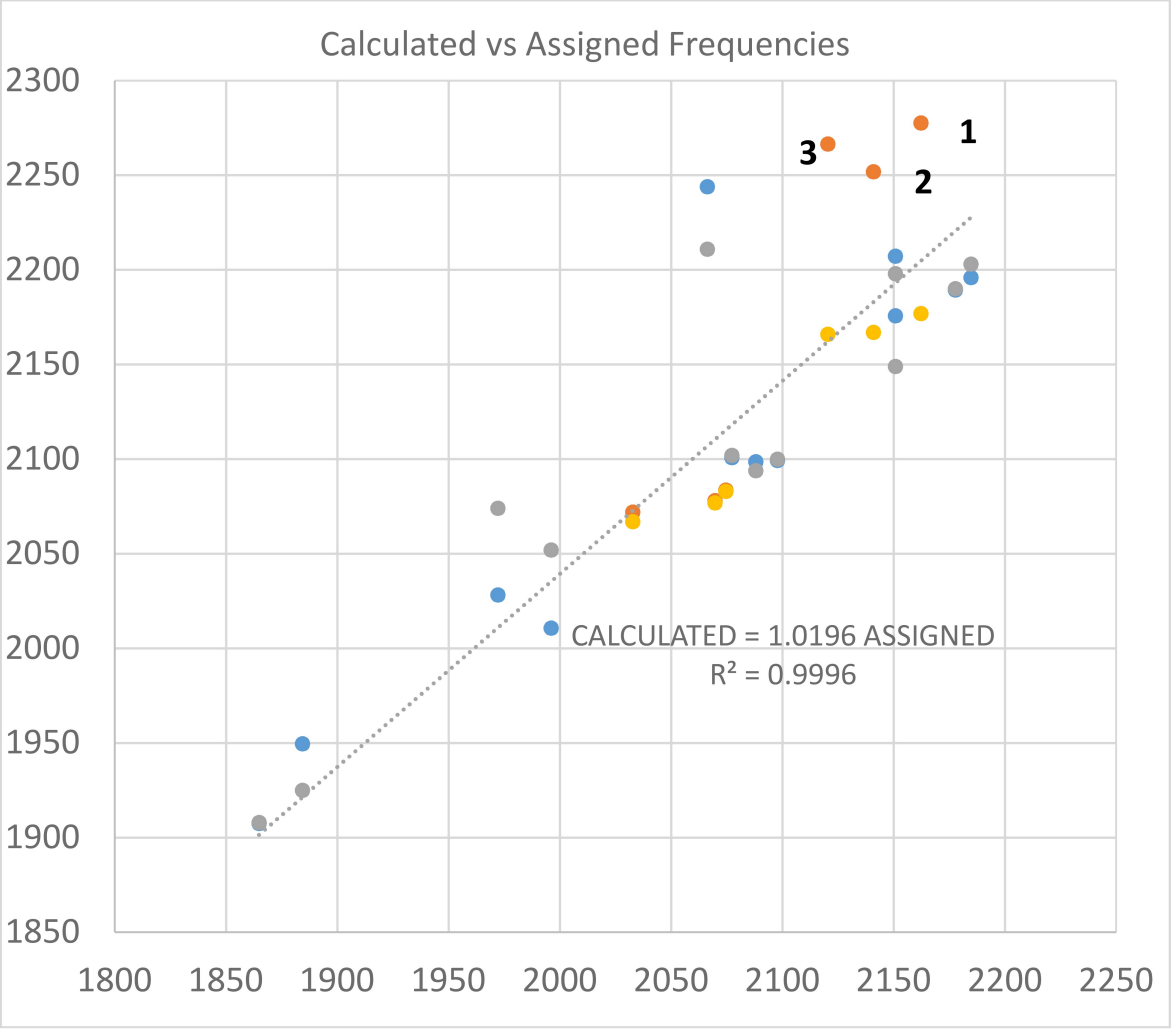
Correlation diagram for calculated (vertical axis) and assigned (horizontal axis) stretching frequencies for cyano, isocyano, and M−H modes. Calculations are conducted with the model CCSD(T)/aug‐cc‐pVTZ with a small pseudopotential (pp) for Cd and Hg. Gaussian calculations are coded orange for triatomics and blue for tetra‐atomics. Molpro calculations are coded yellow for triatomics and gray for tetra‐atomics. Trendline is for Molpro calculated frequencies vs assigned frequencies for tetra‐atomics. Data for this diagram is included in Supplementary Information (Table S3).
  . The trendline suggests a slight overestimate by MOLPRO (<2 %) for tetra‐atomics HM (C, N). Gaussian estimates for MCN are outliers, specifically points **1** (ZnCN), **2** (CdCN) and **3** (HgCN).
6. **Computed frequencies for C−N and M−H stretches, and** for hydrides (Figure 11) show uniform small decreases in value for the sequence H (Zn−Cd−Hg) cyanides. For other systems the pattern is not so simple.
7. **We would like to close with a comparison of the C−N and M−H** stretching frequencies for the molecules investigated here. Figure 12
  **Figure 12:**
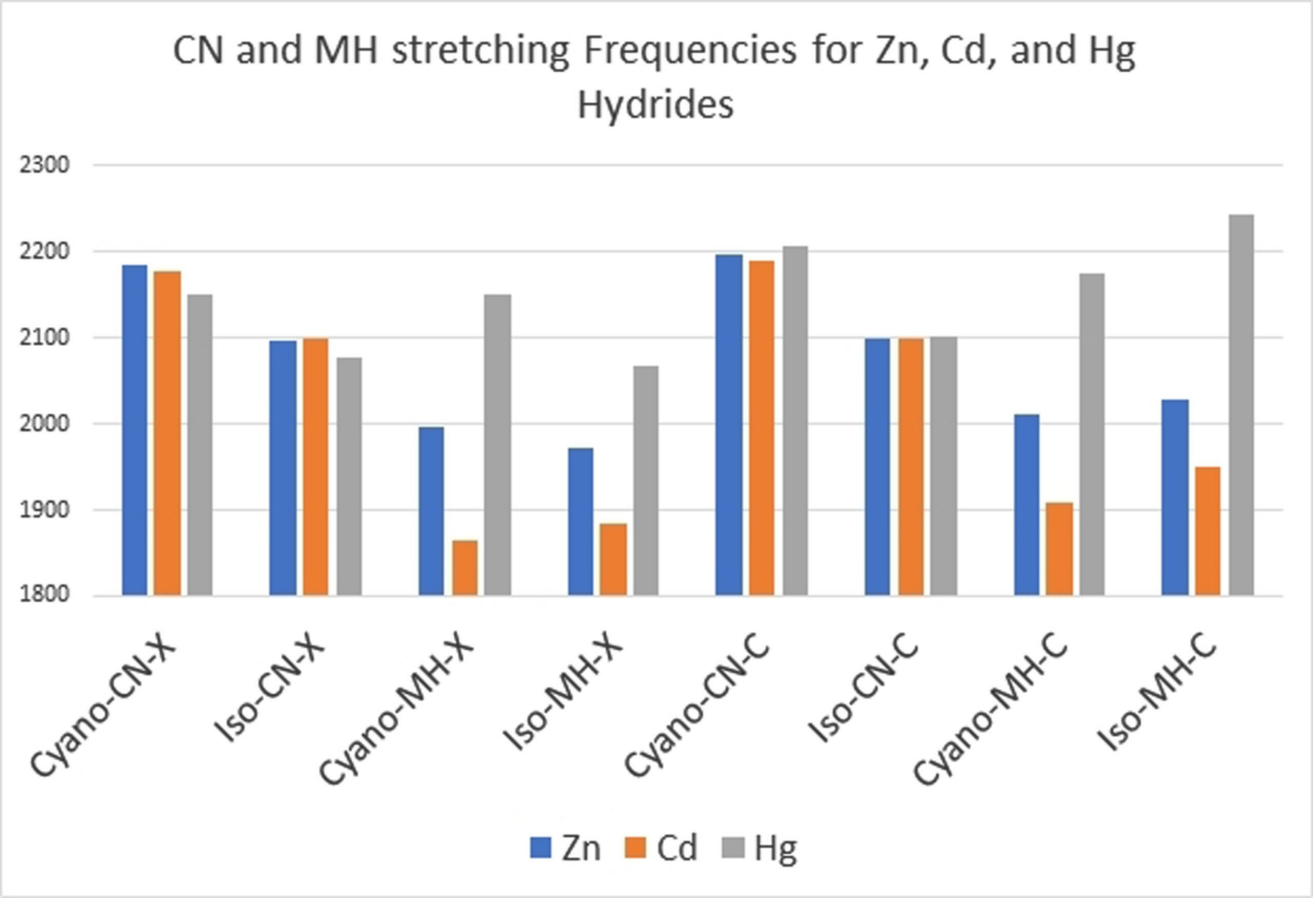
The C−N and M−H stretching frequencies for tetra‐atomic HM(C−N) cyanides and isocyanides, from assignments of experimental data (labeled X) and calculation from Molpro CCSD(T)/aug‐cc‐pVTZ (pp for Cd and Hg) labeled C. The major disagreement is in the H−Hg bond which is surely the most difficult to model.
  depicts those observations.

### 5.5 The ca. 100 cm^−1^ Difference between MCN and MNC Radical Modes (see Table 10)

We observed a nearly constant difference between cyano and isocyano stretching frequencies in a previous study.^[18]^ Summarized in Table 9 are cyano and isocyano frequencies computed by CCSD(T)/aug‐cc‐pVTZ (withpseudo potential for Cd and Hg). The highest frequencies suffer from the erroneously high values for the simple cyanides. These IR‐active modes must be of symmetry σ_u_.

**Table 9:** Cyano and isocyano stretching frequencies from CCSD(T)/aug‐cc‐pVTZ (Zn) and aug‐cc‐pVTZ‐pp (Cd, Hg).[^17^, ^18^]

Cyanide	CN stretch	Isocyanide	CN stretch	Difference
NCZnCN	2202.8	CNZnNC	2098.5	104.3
NCCdCN	2271.7	CNCdNC	2157.0	114.7
NCHgCN	2289.6	CNHgNC	2168.8	120.8

We propose that the roughly 100 cm^−1^ difference between the cyano and isocyano isomer frequencies arises from the distinct differences in charge distributions between cyano and isocyano fragments. The uniform distribution in CN (which accounts for the weak IR absorption of its stretch) can be associated with stronger bonding (reflected in its higher stretching frequency) than the isocyano fragment. The persistence of the 100 cm^−1^ difference suggests that the cyano and isocyano vibrations are not strongly coupled to the other modes of these molecules.

In this work we observe a similar gap near 100 cm^−1^ between HMCN and HMNC, as shown in Table 9. However the CCSD(T) cyano stretches for MCN radicals produce a considerably larger differences (Table 10). This complicated the assignment of those stretches. Fortunately the isotopic ratios allowed us to arrive at an unambiguous assignment, as described in the experimental sections above.

**Table 10:** Collected cyano and isocyano frequencies from CCSD(T)/aug‐cc‐pVTZ (Zn) and aug‐cc‐pVTZ‐pp (Cd, Hg) calculations with Gaussian software.

Cyanide	CN stretch	Isocyanide	CN stretch	Difference
ZnCN	2277.6	ZnNC	2083.6	194.0
CdCN	2251.9	CdNC	2078.1	173.8
HgCN	2266.5	HgNC	2072.1	194.4
HZnCN	2196.0	HZnNC	2099.3	96.7
HCdCN	2189.4	HCdNC	2098.7	90.7
HHgCN	2207.3	HHgNC	2100.9	106.4

An early warning that the MCN system might be exceptional was the ludicrous MP2/aug‐cc‐pVTZ prediction of a CN stretch in ZnCN over 3000 cm^−1^. In the same basis for Zn, MP2 was well behaved for the closed shell hydrides. CCSD and related models (QCISD, BD) gave results consistent with the 100 cm^−1^ rule of thumb. We have no definite explanation for the MCN anomaly. Within Gaussian 09 and 16, a change to Ahlrichs basis sets (def2‐TZVPP) did not alter the anomalies. Oddly, the implementation of CCSD(T) in Molpro produced cyano – isocyano stretching frequency difference (Table 11) near 100 cm^−1^ as expected.

**Table 11:** Cyano and isocyano stretching frequencies by MOLPRO CCSD(T)/aug‐cc‐pVTZ (Zn) and aug‐cc‐pVTZ‐pp (Cd, Hg)

Cyanide	Cyano stretch	Isocyanide	Isocyano stretch	Difference
ZnCN	2177	ZnNC	2083	94
HZnCN	2203	HZnNC	2100	103
CdCN	2167	HCdNC	2077	90
HCdCN	2190	HCdNC	2094	96
HgCN	2166	HgNC	2067	99
HHgCN	2198	HHgNC	2102	96

The Zn−H stretches still elude accurate estimation. CCSD(T)/aug‐cc‐pVTZ frequencies for Gaussian and Molpro are shown in Table 12.

**Table 12:** Collected M−H stretching frequencies calculated by MOLPRO and Gaussian CCSD(T)/aug‐cc‐pVTZ (Zn) and aug‐cc‐pVTZ‐pp (Cd, Hg), with assigned observed frequencies.

Cyanide	H−M	Isocyanide	H−M	Difference	Software
HZnCN	2052	H−ZnNC	2074	22	Molpro
	2008 (2011)		2027 (2028)	19 (17)	Gaussian
	1996		1972		As assigned
HCdCN	1908	HCdNC	1925	17	Molpro
	1907		1949	42	Gaussian
	1865		1884	−19	As assigned
HHgCN	2149	HHgNC	2211	62	Molpro
	2176		2244	68	Gaussian
	1996		1972		As assigned

We observed anomalies in CCSD(T) calculations of cyano stretching frequencies. As a direct result of this major discrepancy CASPT2 (7,6) calculations were conducted. They provided agreement within 20 cm^−1^ and improved the accuracy of the CCSD(T) calculations by fixing the electronic structure to the appropriate configurations.

## 6 Conclusions

Laser ablated Group 12 metals react with hydrogen cyanide to first form the MCN and MNC molecules and then their monohydrides HMCN and HMNC. The ease of hydrogen removal from HCN leads to a high yield of the cyanyl radical, CN, and provides a direct route to the formation of the simple metal cyanides and isocyanides. The hydrides grow markedly on annealing the solid argon matrix to 10, 15 or 20 K, owing to the enhanced mobility of H atoms derived from the reagent HCN in the more porous warmer solid argon host matrix. The radical radical‐reactions are strongly exothermic according to computational modeling. These hydrides were, of course, not produced in earlier metal atom reactions with (CN)_2_.[^17^, ^18^]

Metal isocyanides MNC are less stable (higher energy) than metal cyanides MCN, but the polar charge distribution in the isocyanides gives rise to very intense IR absorptions. These molecules involve three center bonding M−N−C where the central N in M−N−C) moves in an antisymmetric manner between the M and the terminal C or N atom. Previous studies with (CN)_2_ detected these strongly absorbing MNC molecules in the isocyanide spectral region, but not the almost 10‐fold computed weaker absorbing MCN counterparts. The HCN reagent produced increased yields of CN radicals and led to an increased yield of the cyanides, and H atoms for reactions to form the corresponding metal monohydrides.

The structure of solid mercury oxide (chains) differs from that of CdO and ZnO (rock salt). This and the color change from yellow to red for HgO crystals are reported to be relativistic effects.^[36]^

Analogies between the hydride HMCN and HMNC molecules and their parent molecules MCN and MNC aided their identification from matrix infrared spectra with H^13^CN isotopic substitution. Computed vibrational spectra identified intense M−H stretching modes, and −C−N and −N−C stretching modes at higher frequencies than found in other MCN and MNC molecules.

## Conflict of interest

The authors declare no conflict of interest.

## Supporting information

As a service to our authors and readers, this journal provides supporting information supplied by the authors. Such materials are peer reviewed and may be re‐organized for online delivery, but are not copy‐edited or typeset. Technical support issues arising from supporting information (other than missing files) should be addressed to the authors.

Supporting Information
