# Supplementary material for: Cyanides, Isocyanides, and Hydrides of Zn, Cd and Hg from Metal Atom and HCN Reactions: Matrix Infrared Spectra and Electronic Structure Calculations
Source: Chemphyschem. 2021 Aug 13;22(18):1914–34. doi: 10.1002/cphc.202100011 (PMC8518711; doi:10.1002/cphc.202100011)
Supplement: Supplementary file 1 — Supporting Information [file CPHC-22-1914-s001.pdf]

ChemPhysChem

Supporting Information

Cyanides, Isocyanides, and Hydrides of Zn, Cd and Hg from Metal Atom and HCN Reactions: Matrix Infrared Spectra and Electronic Structure Calculations

Hongmin Li, Yetsedaw A. Tsegaw, Lester Andrews,* Carl Trindle, Han-Gook Cho, Tony Stüker, Helmut Beckers, and Sebastian Riedel

SUPPORTING INFORMATION

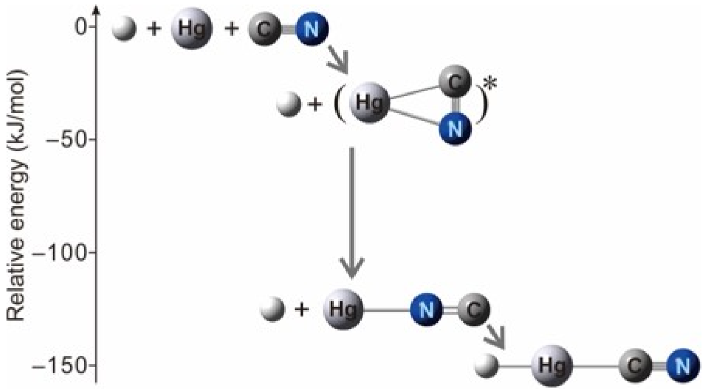

TOC graphic: Outline of the reactions that occur with laser ablated Hg atoms and HCN molecules during condensation in excess argon at 5K and subsequent annealing to allow diffusion and reaction of H atoms with the HgCN and HgNC primary reaction products to form their hydrides.

Picture P-1 Mercury amalgam target photographs

Table S-1 Calculated energies for possible reactions

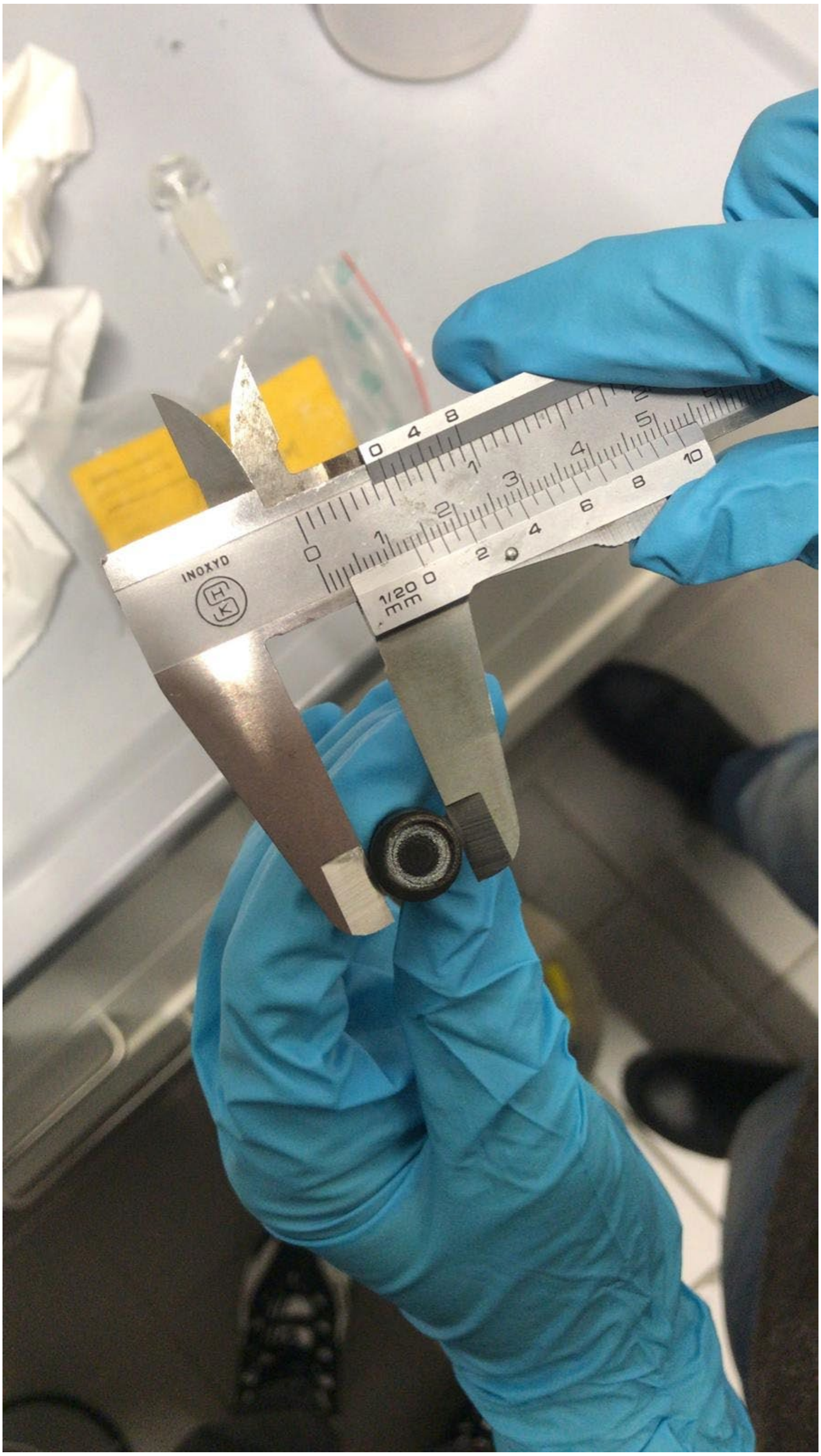

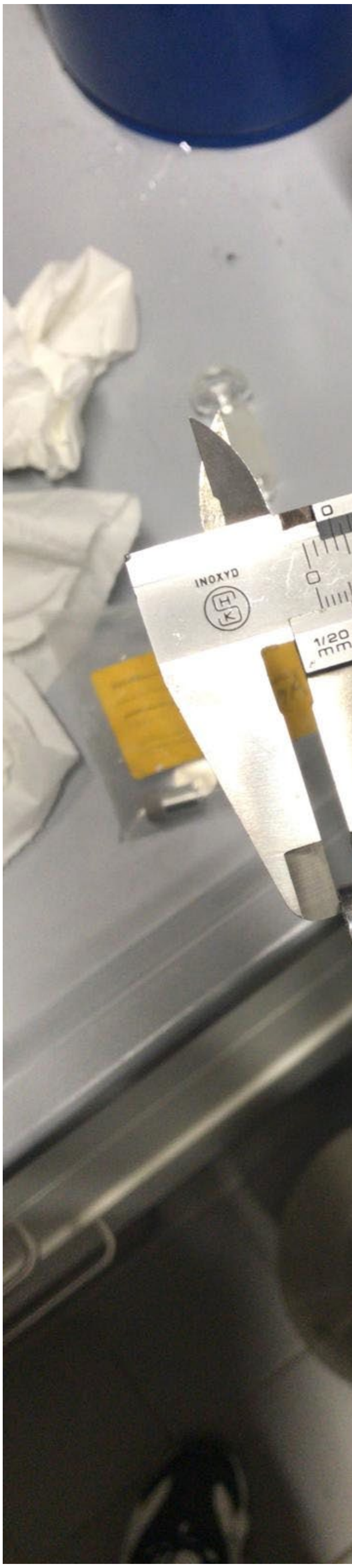

$Zn(^1S_0) + H(^2S_{1/2}) \rightarrow ZnH$	−86.3
$Zn(^1S_0) + ZnH \rightarrow ZnZnH$	−26.5

ZnZnH + H → HZnZnH	−294.7
H + ZnCN → HZnCN	−329.6
H + ZnNC → HZnNC	−420.5
Zn + HZnH → HZnZnH	−5.0
Cd($^1S_0$) → Cd($^3P_0$)	+366.9
Cd($^1S_0$) + CN → CdCN	−200.2
Cd($^1S_0$) + CN → CdNC	−170.9
Cd($^1S_0$) + HCN → HCdCN	+241.4
Cd($^1S_0$) + HCN → HCdNC	+274.1
Cd($^1S_0$) + H($^2S_{1/2}$) → CdH	−64.9
Cd($^1S_0$) + CdH → CdCdH	−20.2
CdCdH + H → HCdCdH	−259.0
H + CdCN → HCdCN	−81.8
H + CdNC → HCdNC	−78.3
Cd + HCdH → HCdCdH	+9.4
Hg($^1S_0$) → Hg($^3P_0$)	+502.7
Hg($^1S_0$) + CN → HgCN	−124.7
Hg($^1S_0$) + CN → HgNC	−69.1
Hg($^1S_0$) + HCN → HHgCN	+374.0
Hg($^1S_0$) + HCN → HHgNC	+411.8
Hg($^1S_0$) + H($^2S_{1/2}$) → HgH	−31.6
Hg($^1S_0$) + HgH → HgHgH	−10.9
HgHgH + H → HHgHgH	−248.6
H + HgCN → HHgCN	−24.7
H + HgNC → HHgNC	−42.5
H + HHgH → HHgHgH	+42.9

Model: CCSD(T) with the aug-cc-pVTZ basis sets are used for H, C, N, and Zn and the aug-cc-pVTZ-pp pseudo potentials and basis sets for Cd and Hg.

MOLPRO electronic-structure calculations for geometry and harmonic vibrations

Computational methods

Density functional theory (DFT) calculations were performed using the TURBOMOLE 7.0.1 program package[1] employing the hybrid exchange-correlation density functional

B3LYP[2] with the polarized triple-ξ basis set def 2-TZVP[3] which applies the Stuttgart-Dresden effective core potential for cadmium and mercury[4]. All Coupled Cluster Single Double and perturbative Triple excitations (CCSD(T)) were carried out using the Dunning's augmented correlation consistent polarized triple-ξ basis sets aug-cc-pVTZ for hydrogen, carbon and nitrogen,[5] as well as aug-cc-pVTZ-PP[6] combined with the effective core potentials[7] ECP10MDF for zinc, ECP28MDF for cadmium and ECP60MDF for mercury. The open and closed shell CCSD(T) calculations were carried out using Molpro 2019.2 in the spin unrestricted RHF-UCCSD(T) open-shell coupled cluster formalism using default frozen core settings.[8] Harmonic vibrational frequency calculations were carried out with all optimized structures analytically (B3LYP) or numerically (CCSD(T)).

Computational results

All MCN, MNC, HMCN and HMNC (M = Zn, Cd and Hg) species converged to linear structures with electronic ground states of $^2\Sigma^+$ (MCN, MNC) and $^1\Sigma_g^+$ (HMCN, HMNC). The obtained structural parameters and harmonic frequencies are given in the supporting information and summarized in Tables S2 and S3.

Table S2: Calculated bond distances for metal cyanides and isocyanides and hydrogenated products

Molecule	d(M-R)	d(C-N)	d(H-M)
HZnCN	193.4 (189.5)	115.5 (116.8)	152.2 (150.0)
HZnNC	183.6 (181.7)	117.2 (118.3)	151.3 (149.1)
HCdCN	208.4 (207.4)	115.5 (116.9))	165.3 (164.9)
HCdNC	202.4 (201.4)	117.1 (118.3)	164.1 (163.9)
HHgCN	206.6 (205.1)	115.4 (116.8)	161.9 (160.7)
HHgNC	202.5 (201.1)	117.1 (118.3)	159.9 (158.6)
ZnCN	198.0 (194.7)	115.6 (117.0)	-
ZnNC	189.4 (186.4)	117.3 (118.5)	-
CdCN	217.4 (214.5)	115.7 (117.0)	-
CdNC	211.4 (207.9)	117.2 (118.4)	-
HgCN	217.0 (213.0)	115.6 (117.0)	-
HgNC	216.4 (209.5)	117.3 (118.5)	-

Calculated M-R (R = C for MCN and HMCN, and N for MNC and HMNC species), C-N and H-M bond lengths (pm) of the linear MCN, MNC, HMCN and HMNC (M = Zn, Cd and Hg) species obtained at the B3LYP and CCSD(T) (parenthesis) levels of theory

Table S3: Calculated vibrational frequencies for metal cyanides and isocyanides and hydrogenated products

Table S 3.

Molecule	ν(CN)	ν(HM)
HZnCN	2286 (26) [2198]	1987 (82) [2052]
HZnNC	2158 (283) [2102]	2005 (60) [2074]
HCdCN	2280 (19) [2190]	1890 (105) [1908]
HCdNC	2157 (273) [2094]	1915 (80) [1925]
HHgCN	2286 (13) [2203]	2115 (100) [2149]
HHgNC	2156 (359) [2100]	2177 (3) [2211]
ZnCN	2264 (27) [2177]	-
ZnNC	2141 (248) [2083]	-
CdCN	2254 (19) [2167]	-
CdNC	2137 (178) [2077]	-
HgCN	2250 (12) [2166]	-

HgNC	2122 (60) [2067]	-

Calculated CN and HM stretching frequencies (cm^{-1}) of HMCN, HMNC, MCN and MNC (M = Zn, Cd and Hg) obtained at the B3LYP and CCSD(T) [brackets] level of theory. Integrated intensities (km mol^{-1}) obtained at the B3LYP level of theory are given in parenthesis.

Table S4: CCSD(T)/aug-cc-pVTZ(-PP) energies of MCN and MNC compounds at their minimum energy geometries

	E (hartree)	ZPE (hartree)	Δ*E* (kJ mol^{-1})	Δ*H* (kJ mol^{-1})
ZnCN	-319.1344796	0.00678392	0	0
ZnNC	-319.1251947	0.00631345	24	23
CdCN	-259.9442502	0.00652252	0	0
CdNC	-259.9322878	0.00599818	31	30
HgCN	-245.5989500	0.00648072	0	0
HgNC	-245.5770061	0.00587129	58	56

Outlook.office.com

Table S5: This is the data for the correlation diagram Fig 11.

	Obs vs Computed				
	Obs	Tetra-G	Tri-G	Tetra-M	Tri-M
HZnC-N	2184.7	2196		2203	
HZnN-C	2097.8	2099.3		2100	
H-ZnCN	1996	2010.8		2052	
H-ZnNC	1972.1	2028.2		2074	
HCdC-N	2177.7	2189.4		2190	
HCdN-C	2088	2098.7		2094	
H-CdCN	1864.8	1907.5		1908	
H-CdNC	1884.3	1949.7		1925	
HHgC-N	**2150.8**	2207.3		2198	
HHgN-C	2077.3	2100.9		2102	
H-HgCN	2150.8	2175.7		2149	
H-HgNC	2066.2	**2243.9**		2211	
ZnC-N	2162.2		**2277.6**		2177
ZnN-C	2074.6		2083.6		2083
CdC-N	2140.9		**2251.9**		2167
CdN-C	2069.7		2078.1		2077
HgC-N	2120.4		**2266.5**		2166

HgN-C	2032.7		2072.1		2067

Table S6: Link between isotopic frequency ratio and displacements in normal modes (CCSD/aug-cc-pVTZ)

Species	12/13 ratio	14/15 ratio	Qterm	Qcentral	Qmetal	QH	frequency
**$Zn^{12}C^{14}N$**			**-0.63 (N)**	**0.78 (C)**	**-0.01**		**2260.0**
	1.02267		-0.66 (N)	0.75 (C)	-0.01		2209.9
**$Zn^{14}N^{12}C$**			**-0.74 (C)**	**0.68 (N)**	**-0.01**		**2148.2**
	1.02004		-0.71 (C)	0.71 (N)	-0.01		2106.0
		1.01656	-0.76 (C)	0.65 (N)	-0.01		2113.2
**$HZn^{12}C^{14}N$**			**-0.62**	**0.78**	**-0.01**	**0.04**	**2265.3**
	1.02248		-0.65	0.76	-0.01	0.05	2215.5
		1.01401	-0.59	0.80	-0.01	0.04	2234.0
$Cd^{12}C^{14}N$							
**$Cd^{14}N^{12}C$**			**-0.74 (C)**	**0.67 (N)**	**0.00**		**2244.8**
	1.02083		-0.71 (C)	0.70 (N)	0.00		2199.0
		1.01588	-0.76 (C)	0.64 (N)	0.00		2209.7
**$HCd^{12}C^{14}N$**			**-0.62 (N)**	**0.78 (C)**	**-0.01**	**0.02**	**2261.3**
	1.02280		-0.66 (N)	0.75 (C)	-0.01	0.03	2210.9
		1.01454	-0.60 (N)	0.80 (C)	-0.01	0.02	2228.9
**$HCd^{14}N\ ^{12}C$**			**-0.74 (C)**	**0.68 (N)**	**-0.01**	**0.03**	**2159.2**
			-0.71 (C)	0.71 (N)	-0.01	0.05	2117.0
			-0.76 (C)	0.65 (N)	-0.01	0.04	2133.3
**$Hg^{12}C^{14}N$**			**-0.63 (N)**	**0.78 (C)**	**0.00**		**2264.8**
	1.02253		-0.66 (N)	0.75 (C)	0.00		2214.9
		1.01474	-0.60 (N)	0.80 (C)	0.00		2231.9
**$Hg\ ^{14}N^{12}C$**			**-0.74 (C)**	**0.67 (N)**	**0.00**		**2139.7**
	1.02026		-0.71 (C)	0.70 (N)	0.00		2097.2
		1.01668	-0.76 (C)	0.65 (N)	0.00		2104.6
$HHg^{12}C^{14}N$							
$HHg\ ^{14}N^{12}C$							

Ratios are defined by frequencies (rightmost column). Note when the 12/13 ratio decreases, the 14/15 ratio increases

Table S7: Link between isotopic frequency ratio and displacements in normal modes (ωB97XD/def2tzvp)

Species	12/13 ratio	14/15 ratio	Qterm	Qcentral	QM	QH	Freq
HZnCN	1.02293	1.01511	0.624 (N)	-0.781 (C)	-0.010	0.019	2329
HZnNC	1.01966	1.01707	0.734 (C)	-0.679 (N)	0.011	0.005	2189
ZnCN	1.02313	1.01511	-0.630 (N)	0.777 (C)	-0.008		2310
ZnNC	1.01992	1.01689	-0.737 (C)	0.675 (N)	-0.010		2178
HCdCN			-0.625 (N)	0.781 (C)	-0.005	0.009	2325
HCdNC			-0.736 (C)	0.677 (N)	-0.006	0.017	2190
CdCN			-0.631 (N)	0.776 (C)	-0.130		2302
CdNC			-0.746 (C)	0.666 (N)	-0.003		2170
HHgCN			-0.614 (N)	0.765 (C)	-0.004	0.194	2336
HHgNC			-0.730 (C)	-0.677 (N)	-0.004	0.097	1297
HgCN			-0.629 (N)	0.769 (C)	-0.002		
HgNC			0.743 (C)	-0.669 (N)	0.002		

Ratios are defined by frequencies (rightmost column). Note when the 12/13 ratio decreases, the 14/15 ratio increases

Figures S1-S4

Table S8

Molecule	Metal-(C,N)	C-N	H-M
ZnCN	196.7 (194.7)[a]	116.2 (117.0)[b]	
ZnNC	187.8 (186.4)[c]	117.7 (118.5)[d]	
HZnCN	1931.6 (189.5)[c]	116.1 (116.8)[d]	152.0 (150.0)[e]
HZnNC	183.4 (181.7)	117.6 (116.9)	151.2 (149.1)

Microwave data, Ref 23: (a) 195.4, (b) 114.2; Ref 24: (c) 189.7, (d) 114.6, (e) 149.5 pm

Molecule	Metal-(C,N)	C-N	H-M
CdCN	214.6 (214.5)	116.2 (117.0)	
CdNC	207.8 (207.9)	117.7 (118.4)	
HCdCN	207.7 (207.4)	116.1 (116.9)	165.1 (164.9)
HCdNC	201.4 (201.4)	117.6 (118.3)	164.0 (163.9)

Molecule	Metal-(C,N)	C-N	H-M
HgCN	211.9 (213.0)	116.1 (117.0)	
HgNC	207.9 (209.5)	117.7 (118.5)	
HHgCN	204.6 (205.1)	115.9 (116.8)	160.1 (160.7)
HHgNC	200.3 (201.4)	117.5 (118.3)	158.1 (158.6)

CCSD values, with CCSD(T) counterparts in brackets; distance in picometers

Molecule	Metal-(C,N)	C-N	H-M
ZnCN	196.7 (194.7)[a]	116.2 (117.0)[b]	
ZnNC	187.8 (186.4)[c]	117.7 (118.5)[d]	
HZnCN	1931.6 (189.5)[c]	116.1 (116.8)[d]	152.0 (150.0)[e]
HZnNC	183.4 (181.7)	117.6 (116.9)	151.2 (149.1)

Microwave data, Ref 23: (a) 195.4, (b) 114.2; Ref 24: (c) 189.7, (d) 114.6, (e) 149.5 pm

Molecule	Metal-(C,N)	C-N	H-M
CdCN	214.6 (214.5)	116.2 (117.0)	
CdNC	207.8 (207.9)	117.7 (118.4)	
HCdCN	207.7 (207.4)	116.1 (116.9)	165.1 (164.9)
HCdNC	201.4 (201.4)	117.6 (118.3)	164.0 (163.9)

Molecule	Metal-(C,N)	C-N	H-M
HgCN	211.9 (213.0)	116.1 (117.0)	
HgNC	207.9 (209.5)	117.7 (118.5)	
HHgCN	204.6 (205.1)	115.9 (116.8)	160.1 (160.7)
HHgNC	200.3 (201.4)	117.5 (118.3)	158.1 (158.6)

CCSD values, with CCSD(T) counterparts in brackets; distance in picometers

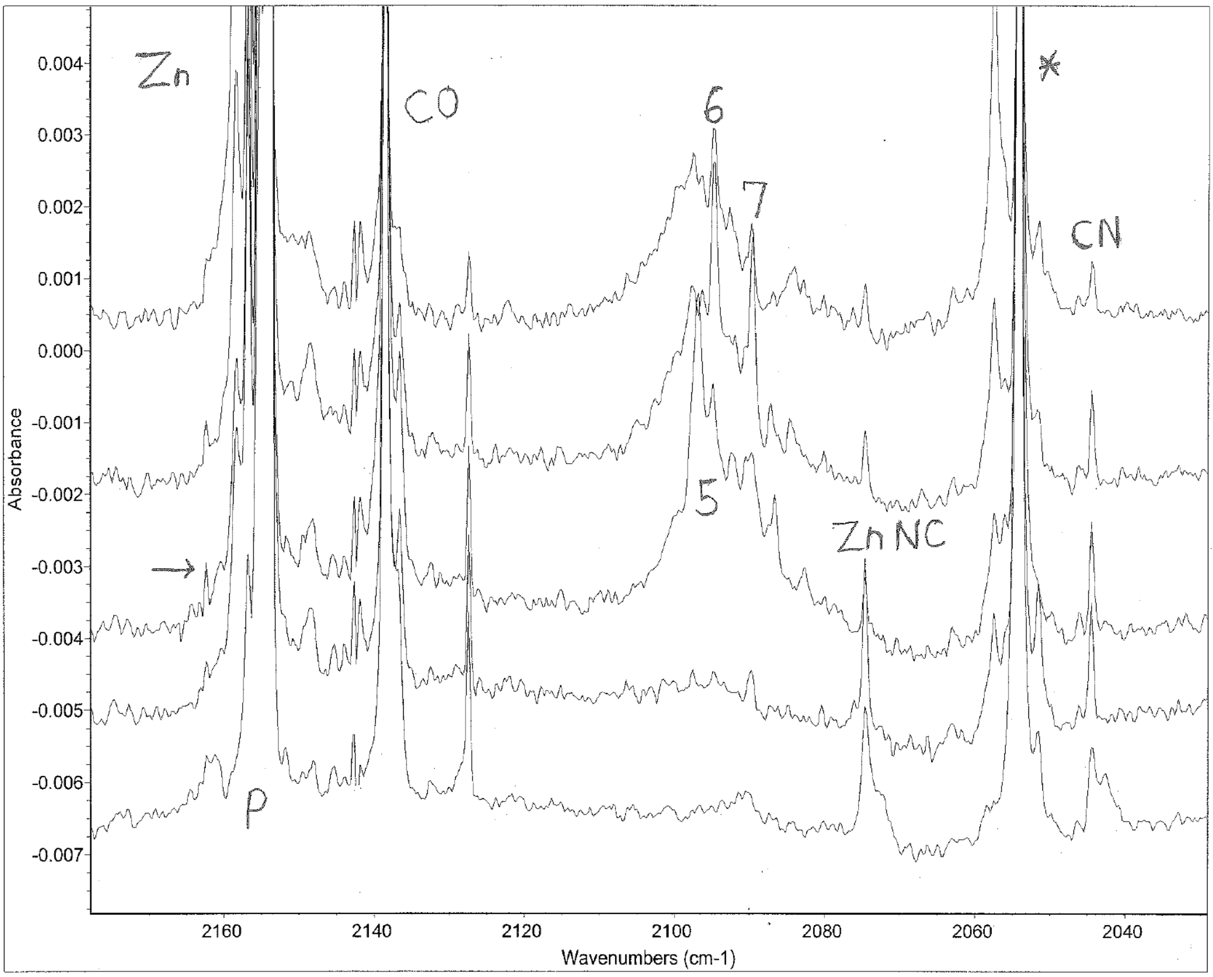

Figure S1: Infrared spectra using Nicolet iS50 FTIR for the products of laser ablated zinc atom reactions with cyanogen in excess argon at 4 K. Bottom spectrum recorded following deposition of Zn with 1% $(CN)_2$ for one hour: The arrow points to the ZnCN product absorption measured at 2162.4 cm^{-1}, P denotes the major $(CN)_2$ absorption: the sharp 2127.6 cm^{-1} band is from $N^{12}C^{13}CN$ in natural abundance. The numbers 5, 6, 7 are for major products listed in the observed frequency table. The * indicates the strongest CNCN precursor isomer absorption. The next spectrum going up follows annealing to 25 K, and the next was recorded after full mercury arc irradiation for 10 min: note the substantial growth in the major products labeled 5, 6, and 7. The last two scans going up were recorded after annealing to 30 and 40 K where 6 and 7 increase at the expense of 5.

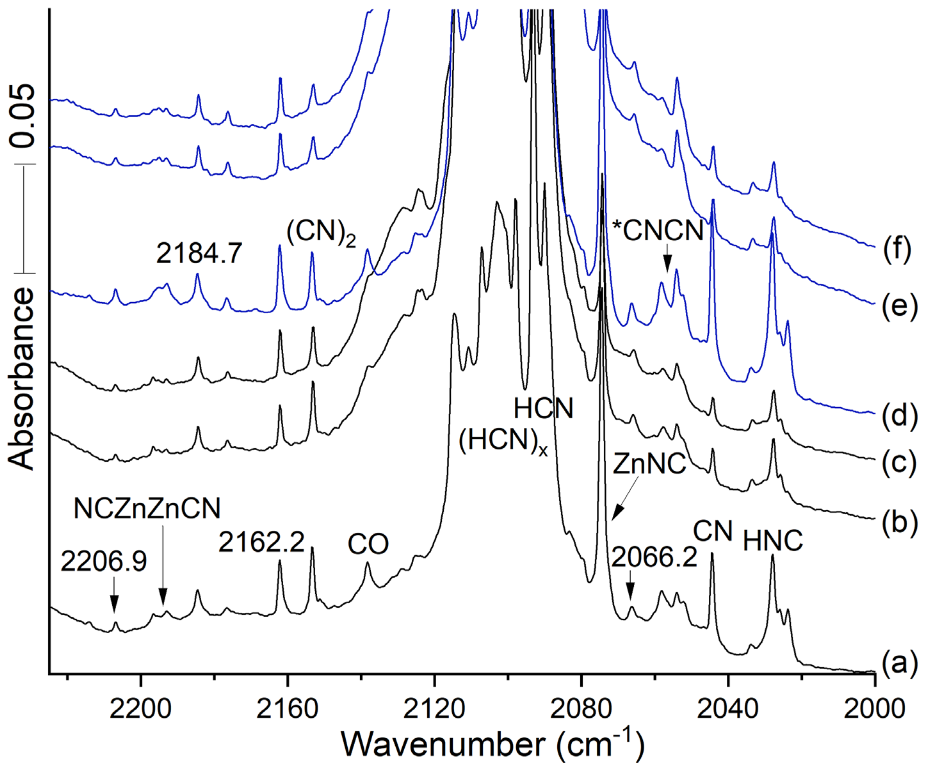

Figure S2. Infrared spectra of the reaction products from laser ablated Zn co-deposited with 1% HCN argon at 5 K. Spectrum after (a) deposition for 120 min with regular laser energy (50% of maximum energy), (b) annealing to 20 K and cooling back to 5 K, (c) full arc irradiation for 20 min for black spectra (d) deposition for 120 min with 20% higher laser energy (60% of maximum energy), (e) annealing to 20 K and cooling back to 5 K, (f) full arc irradiation for 20 min for blue spectra.

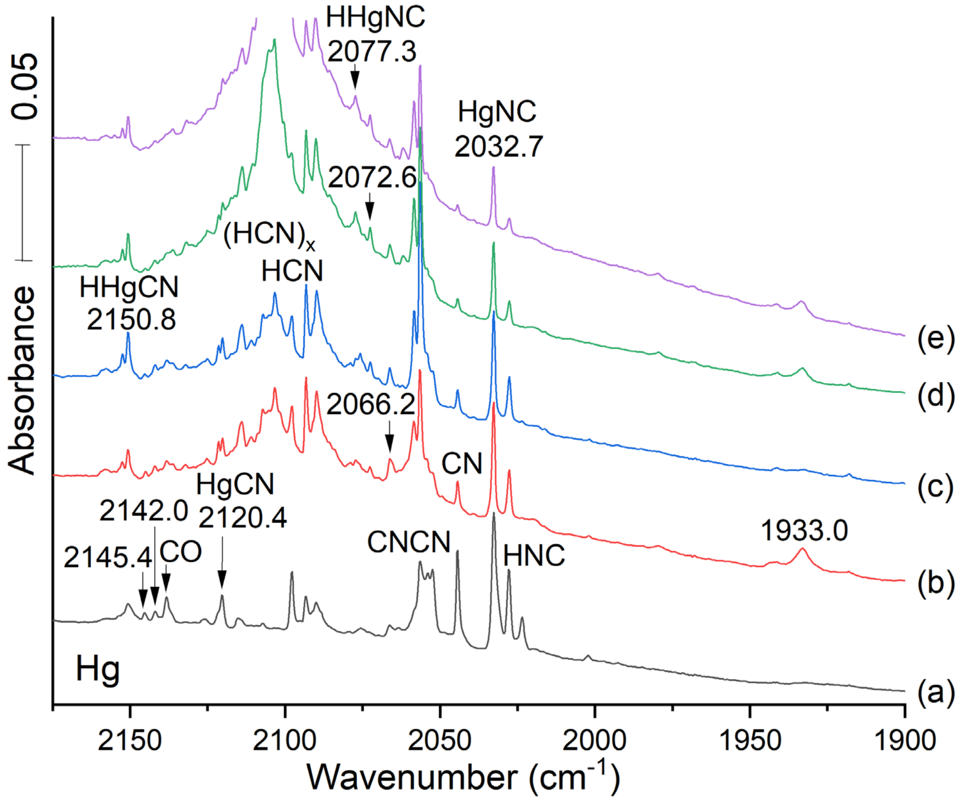

Figure S3. Infrared Spectra of the laser ablated Hg atom reaction products with 0.2% HCN in Argon during Co-deposition at 5 K. Black spectrum (a) recorded after sample deposition for 120 min. Red spectrum (b) after annealing to 20 K and re-cooling to 5 K. Blue spectrum (c) after Hg arc photolysis for 20 min. Green spectrum (d) after annealing to 30 K, and Purple spectrum (e) after annealing to 35 K.

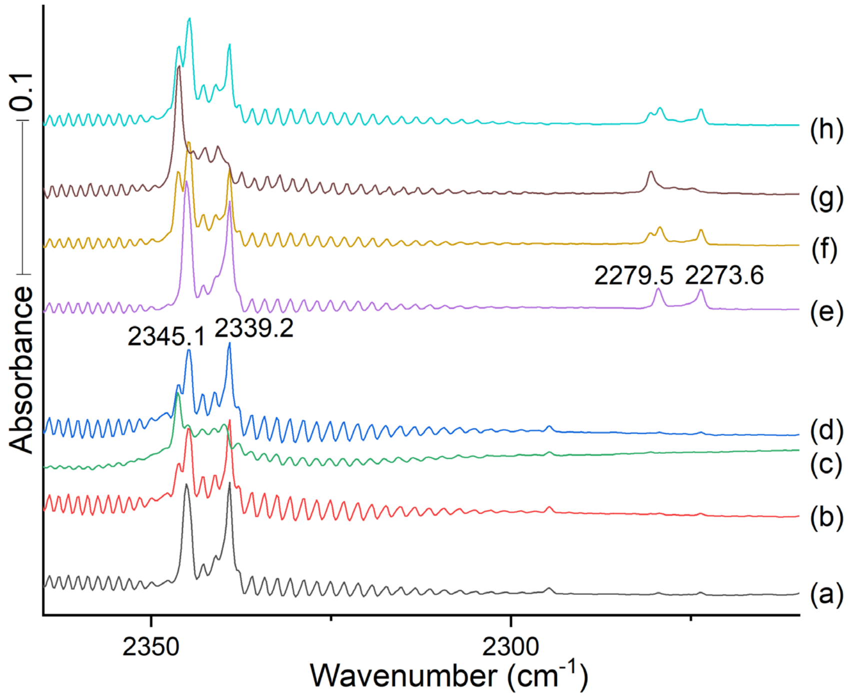

Figure S4. Infrared spectra between 2360 and 2260 cm^{-1} for the natural isotopic carbon dioxide trace impurity and from the reaction of C atoms with trace O_2 impurity and the $O^{13}CO$ reaction product (lower frequency doublet) from laser ablated Hg co-deposited with 0.2% HCN (a-d) or with 0.2% $H^{13}CN$ (e-h) in argon at 5 K, respectively. The HOO radical was detected at 1388 cm^{-1} so we know that trace air impurity contributed to the production of $O^{12}CO$ and $O^{13}CO$. Spectrum after (a) and (e) deposition for 120 min, (b) and (f) annealing to 20 K and cooling back to 5 K, (c) and (g) full Hg arc Photolysis for 20 min, (d) and (h) after annealing to 35 K and cooling back to 5 K. Numbers are given for matrix site splittings in the antisymmetric stretching mode for $O^{12}CO$ and $O^{13}CO$.

(Information used for calculations of product bond lengths, energies and vibrational frequencies given in Supporting Information Section)

(1) TURBOMOLE GmbH, TURBOMOLE V7.0.1, 2015.
(2) a) P J. Stephens, F. J. Devlin, C. F. Chabalowski, M. J. Frisch, J. Phys. Chem. 1994, 98, 11623; b) S H. Vosko, L. Wilk, M. Nusair, Can. J. Phys. 1980, 58, 1200; c) C. Lee, W. Yang, R. G. Parr, Phys. Rev. B 1988, 37, 785; d) A. D. Becke, J. Chem. Phys. 1993, 98, 5648.
(3) F. Weigend, R. Ahlrichs, Phys. Chem. Chem. Phys. 2005, 7, 3297.
(4) D. Andrae, U. Huermann, M. Doig, H. Stoll, H. Preu, Theor. Chim. Acta 1990, 77, 123.
(5) a) R. A. Kendall, T. H. Dunning, R. J. Harrison, J. Chem. Phys. 1992, 96, 6796; b) T. H. Dunning, J. Chem. Phys. 1989, 90, 1007.[6] K. A. Peterson, C. Puzzarini, Theor. Chem. Acc. 2005, 114, 283.
(7) D. Figgen, G. Rauhut, M. Doig, H. Stoll, Chem. Phys. 2005, 311,227.
(8) H.-J. Werner, P. J. Knowles, G. Knizia, F. R. Manby, M. Schutz, WIREs Comput Mot Sci 2012, 2, 242, F. R. Manby, M. Schutz, P. Celani, W. Gyorffy, D. Kats, T. Korona, R. Lindh, A. Mitrush_enkov, G. Rauhut, K. R. Shamasundar, T. B. Adler, R. D. Amos, S. J. Bennie, A. Bernhardsson, A. Berning, D. L. Cooper, M. J. 0. Deegan, A. J. Dobbyn, F. Eckert, E. Goll, C. Hampel, A. Hesselmann, G. Hetzer, T. Hrenar, G. Jansen, C. Koppl, S. J. R. Lee, Y. Liu, A. W. Lloyd, Q. Ma, R. A. Mata, A. J. May, S. J. McNicholas, W. Meyer, T. F. Miller Ill, M. E. Mura, A. Nicklass, D. P. O'Neill, P. Palmieri, D. Peng, K. Pfluger, R. Pitzer, M. Reiher, T. Shiozaki, H. Stoll, A. J. Stone, R. Tar Welborn, MOLPRO, version 2019.2, a package of ab initio programs; c) P. J. Knowles, C. Hampel, H.-J. Werner, J. Chem. Phys. 1993, 99, 5219.

Table S9 Bond lengths for cyanides and isocyanides MOLPRO/ aug-cc-pVTZ (Zn, C, N) and aug-cc-pVTZ-pp (Cd, Hg)

Molecule	Metal-(C,N)	C-N	H-M
ZnCN	196.7 (194.7)[a]	116.2 (117.0)[b]	
ZnNC	187.8 (186.4)[c]	117.7 (118.5)[d]	
HZnCN	1931.6 (189.5)[c]	116.1 (116.8)[d]	152.0 (150.0)[e]
HZnNC	183.4 (181.7)	117.6 (116.9)	151.2 (149.1)

Microwave data, Ref 23: (a) 195.4, (b) 114.2; Ref 24: (c) 189.7, (d) 114.6, (e) 149.5 pm

Molecule	Metal-(C,N)	C-N	H-M
CdCN	214.6 (214.5)	116.2 (117.0)	
CdNC	207.8 (207.9)	117.7 (118.4)	
HCdCN	207.7 (207.4)	116.1 (116.9)	165.1 (164.9)
HCdNC	201.4 (201.4)	117.6 (118.3)	164.0 (163.9)

Molecule	Metal-(C,N)	C-N	H-M
HgCN	211.9 (213.0)	116.1 (117.0)	
HgNC	207.9 (209.5)	117.7 (118.5)	
HHgCN	204.6 (205.1)	115.9 (116.8)	160.1 (160.7)
HHgNC	200.3 (201.4)	117.5 (118.3)	158.1 (158.6)
